# Seminal plasma induces inflammation and enhances HIV-1 replication in human cervical tissue explants

**DOI:** 10.1371/journal.ppat.1006402

**Published:** 2017-05-19

**Authors:** Andrea Introini, Stéphanie Boström, Frideborg Bradley, Anna Gibbs, Axel Glaessgen, Annelie Tjernlund, Kristina Broliden

**Affiliations:** 1 Unit of Infectious Diseases, Center for Molecular Medicine, Department of Medicine Solna, Karolinska University Hospital Solna, Karolinska Institutet, Stockholm, Sweden; 2 Department of Clinical Pathology and Cytology, Unilabs AB, Capio St Göran Hospital, Stockholm, Sweden; Emory University, UNITED STATES

## Abstract

The most immediate and evident effect of mucosal exposure to semen *in vivo* is a local release of proinflammatory mediators accompanied by an influx of leukocytes into the female genital mucosa (FGM). The implication of such response in HIV-1 transmission has never been addressed due to limitations of currently available experimental models. Using human tissue explants from the uterine cervix, we developed a system of mucosal exposure to seminal plasma (SP) that supports HIV-1 replication. Treatment of ectocervical explants with SP resulted in the upregulation of inflammatory and growth factors, including IL-6, TNF, CCL5, CCL20, CXCL1, and CXCL8, and *IL1A*, *CSF2*, *IL7*, *PTGS2*, as evaluated by measuring protein levels in explant conditioned medium (ECM) and gene expression in tissue. SP treatment was also associated with increased recruitment of monocytes and neutrophils, as observed upon incubation of peripheral blood leukocytes with ECM in a transwell system. To evaluate the impact of the SP-mediated response on local susceptibility to HIV-1, we infected ectocervical explants with the CCR5-tropic variant HIV-1_BaL_ either in the presence of SP, or after explant pre-incubation with SP. In both experimental settings SP enhanced virus replication as evaluated by HIV-1 p24_gag_ released in explant culture medium over time, as well as by HIV-1 DNA quantification in explants infected in the presence of SP. These results suggest that a sustained inflammatory response elicited by SP soon after coitus may promote HIV-1 transmission to the FGM. Nevertheless, ectocervical tissue explants did not support the replication of transmitted/founder HIV-1 molecular clones, regardless of SP treatment. Our system offers experimental and analytical advantages over traditional models of HIV-1 transmission for the study of SP immunoregulatory effect on the FGM, and may provide a useful platform to ultimately identify new determinants of HIV-1 infection at this site.

## Author summary

The majority of HIV-1 transmissions worldwide occur via unprotected vaginal intercourse, with semen and the female genital mucosa (FGM) being the main vector and recipient of virus acquisition in women. It is well known that mucosal exposure to semen via vaginal intercourse or artificial fertilization results in transient inflammation of the FGM. This response is thought to be important to defend the host against eventual pathogens present in semen and clear the mucosa from residual sperm cells, as well as to promote fertilization. However its implication in HIV-1 transmission has never been addressed. Using human tissue explants from the uterine cervix, we developed a model of mucosal exposure to seminal plasma (SP) to study HIV-1 transmission to the FGM. Our model recapitulated the main features of the *in vivo* response to semen, namely upregulation of proinflammatory factors and leukocyte recruitment. SP treatment of explants enhanced HIV-1 replication, suggesting that a sustained inflammatory response elicited by SP soon after coitus may promote virus infection. Our experimental system may contribute to improve the efficacy of currently available prevention measures by understanding the mechanisms regulating immunity in the FGM, and leading to the identification of new determinants of HIV-1 transmission therein.

## Introduction

Mucosal exposure to infected semen accounts for the majority of human immunodeficiency virus (HIV) 1 transmission events worldwide, with vaginal intercourse being the most common acquisition route in women [1]. In spite of this evidence, a poor understanding of the mechanisms regulating immunity at mucosal sites, exacerbated by discrepancies in the biology of infection between humans and experimental models, has hampered the development of effective prevention measures against HIV-1 [2–4].

We among others believe that semen plays an active role in modulating the efficiency of HIV-1 transmission to the female genital mucosa (FGM) [5]. Semen is a complex mixture of cells and molecules with immunoregulatory function [6]. An inflammatory response characterized by leukocyte infiltration and upregulation of proinflammatory and growth factors occurs in the FGM soon after exposure to semen, in a highly conserved fashion among mammals [7]. This response, also called leukocytic reaction, was originally described in humans by analyzing cervical smears collected after artificial insemination, showing a significant amount of neutrophils that egressed the mucosa as early as 20 min after treatment [8,9]. These events have been proposed to contribute to fertilization, nevertheless their impact on the FGM susceptibility to sexually transmitted pathogens, including HIV-1, remains elusive.

In the last few years, a growing number of reports investigated the role of semen in HIV-1 infection leading to controversial results [5,10]. Most of these findings rely on isolated cells incubated with highly diluted seminal plasma (SP) and artificial infection settings involving exogenously activated or reporter cells, which may not adequately mimic the biology of mucosal transmission. The few vaginal infection studies conducted to date in non-human primates employed either purified seminal factors or human SP, without assessing the presence of an underlying immune response induced by SP treatment [11,12].

To develop a new, relevant model of HIV-1 sexual transmission we used human mucosal tissue from the lower female reproductive tract (FRT) and devised an experimental system that could reproduce the local immunologic events associated with exposure to semen as observed *in vivo*. We here demonstrate that treatment of ectocervical tissue explants with SP from HIV-seronegative men recapitulated the hallmarks of the early mucosal response to semen, including proinflammatory and growth factor upregulation, as well as leukocyte recruitment as evaluated in a transwell system. This inflammatory-like response resulted in enhanced HIV-1 replication, as shown in explants infected with the laboratory-adapted CCR5-tropic HIV-1 variant HIV-1_BaL_ both after exposure to SP and in the presence of SP. However, our system did not support the replication of transmitted/founder HIV-1 molecular clones, regardless of SP treatment. Exposure to SP did not affect cell viability as evaluated on individual cells upon isolation from tissue explants, that were mostly viable after a 24 h-culture in agarose. Tissue architecture was partially preserved after 18 days from infection, allowing for the detection and visualization of cells harboring HIV-1 within explants at the end of culture. These aspects highlight the relevance and advantages offered by tissue explants over other *in vitro* models of mucosal response to semen and HIV-1 transmission. In particular, our experimental system can be implemented to gain a better understanding of the cellular and molecular features of SP early immunoregulatory effect on the FGM. Such knowledge may contribute to improve the efficiency of currently available prevention strategies by enabling the targeting of other determinants of transmission in addition to HIV-1.

## Results

### Tissue donors

Specimens from 54 uterine tissue donors were included. Median donor age (range) was 45 (35–54) years. All women were pre-menopausal. Fourteen women were using progesterone-based medications, namely levonorgestrel intrauterine device (IUD) or the selective progesterone receptor modulator ulipristal acetate or both, at the time of surgery: specimens from one donor were used for cytokine analysis, three for cell viability assay, and ten for HIV-1 infection experiments, of which six were infected with HIV-1_BaL_. Seventeen donors provided information on sexual habits: all donors reported one sexual partner in the past year, and two reported regular condom use.

### SP upregulates proinflammatory and growth factors in ectocervical tissue explants

#### a) Cytokine concentrations in culture medium

To assess whether *ex vivo* exposure of the FGM to SP triggers a local inflammatory response such as that following coitus, we measured the levels of selected inflammation markers and growth factors released into the culture supernatant of donor-matched ectocervical tissue explants (designated explant conditioned medium [ECM]) incubated for 2, 4, and 12 h with SP from healthy donors at the concentration of 50% and 25%, or with culture medium (CM) alone as a control (S1A Fig). The inflammatory regulators interleukin (IL)-1α, IL-6, tumor necrosis factor α (TNF), transforming growth factor (TGF)-β1 and IL-10, and the chemokines C-C motif chemokine (CCL) 5 (RANTES), CCL20 (MIP-3α), C-X-C motif chemokine (CXCL) 1 (GRO-α) and CXCL8 (IL-8) were screened based on previously reported data on cytokine responses elicited by semen *in vivo* and *in vitro* [7,13].

**Fig 1:**
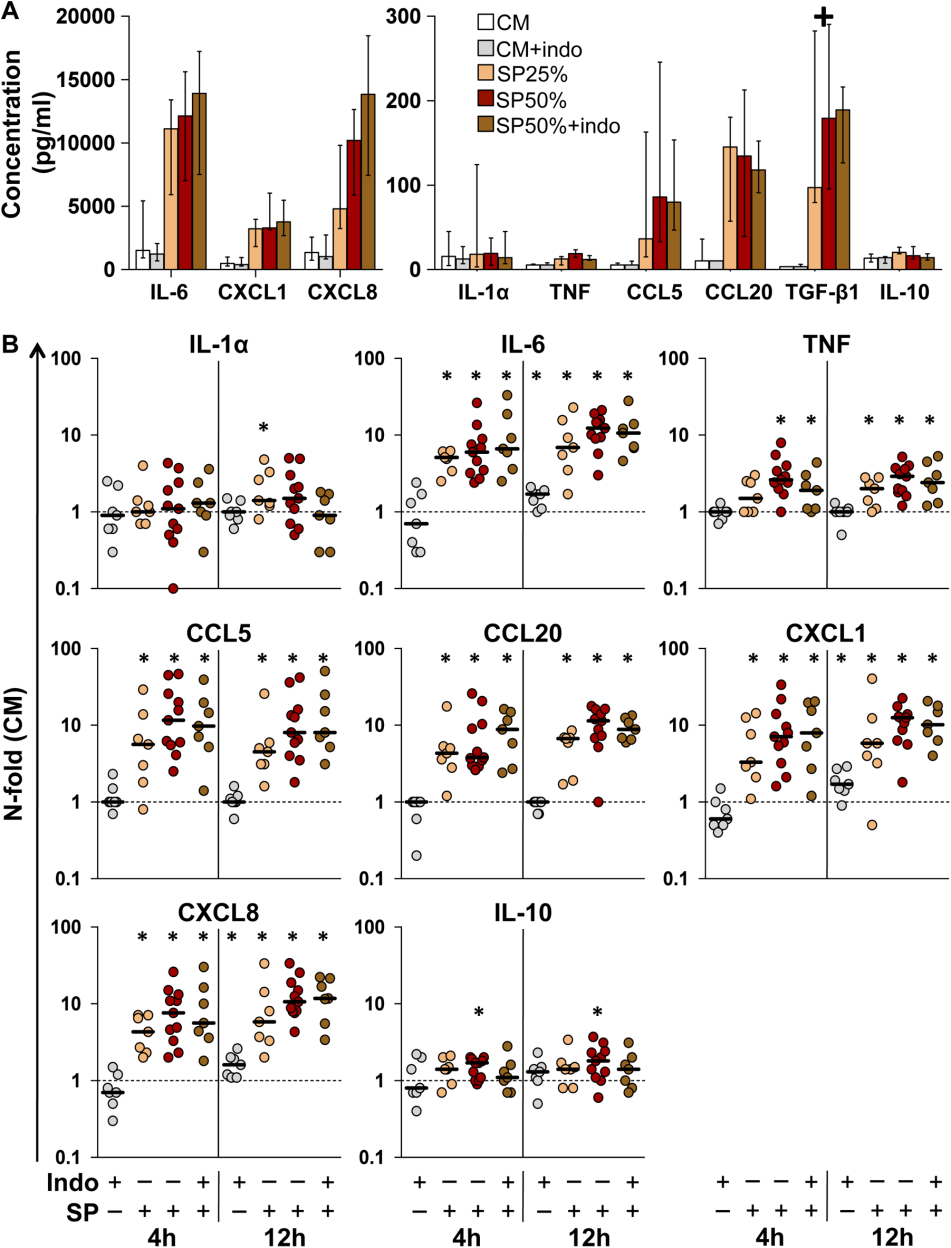
Cytokine concentration in ectocervical tissue explant conditioned medium (ECM). **A)** Cytokine concentration (pg/ml) was measured in ECM of ectocervical explants incubated with culture medium (CM), seminal plasma (SP) 25% or SP50% in the presence or absence of indomethacin (indo) 10μM for 4 h, followed by an additional 12 h-incubation with medium only. Bars represent median with interquartile range (IQR). + excluded from the analysis (TGF-β1). **B)** N-fold change in ECM cytokine concentration of explants treated with SP and/or indomethacin for 4 or 12 h, compared to donor-matched explants incubated with CM. Bars indicate median values. Asterisks denote a statistically significant difference with CM (Wilcoxon signed rank test, p<0.05). CM (white, n = 11), CM+indomethacin (gray, n = 7), SP25% (orange, n = 7), SP50% (red, n = 11), and SP50%+indomethacin (brown, n = 7).

In ECM of untreated control explants (i.e., CM), IL-6, CXCL1, and CXCL8 exhibited the highest levels, whereas TNF and TGF-β1 were undetectable for most donors at all time points (Fig 1A and S1 Table). This profile reflected the cytokine levels measured in genital secretions of healthy women [14,15]. However, the secretions collected in the vagina likely originate from multiple sites across the FRT, and in our system cytokine production can be affected by tissue repair processes induced by mechanical damage inflicted during dissection and/or hypoxia during culture [16], as demonstrated by IL-6 levels that were higher in ECM than in genital secretions of healthy women.

Based on preliminary observations, we set arbitrary thresholds of cytokine concentration to define the preinflammatory status of tissue specimens. Experiments in which ECM of untreated control explants exhibited IL-6 or CXCL8 levels >10 ng/ml for any incubation time were excluded from the analysis (n = 8). In seven of these experiments, treatment with the cyclooxygenase-inhibitor indomethacin significantly reduced the release of both IL-6 (p = 0.022) and CXCL8 (p = 0.015) (S2 Fig). Although the immunoregulatory function of sex hormones on the lower FRT has not been investigated as thoroughly as their effect on the upper FRT, by excluding experiments in which ectocervical explants produced high levels of CXCL8 we might have selected donors outside the perimenstrual stage of the cycle. This stage is indeed characterized by prostaglandin-mediated induction of CXCL8, which was shown to be attenuated *ex vivo* by indomethacin treatment in human endometrial tissue [17]. Unfortunately, it was not possible to estimate the tissue donors’ menstrual-cycle stage through the questionnaire due to frequent/irregular bleeding associated with their medical condition. Multiple additional factors can induce prostaglandin production in the lower FRT, such as hygienic practices, microfora composition, and asymptomatic viral infections [18,19], but we were not able to address such factors in our cohort of tissue donors to set more stringent inclusion criteria. In included experiments, when present, indomethacin did not significantly affect the baseline levels of all 9 examined cytokines, except for an increase (median) of IL-6 (1.7-fold), CXCL1 (1.7-fold), and CXCL8 (1.6-fold) after 12 h of incubation (n = 7, p<0.05) (Fig 1B and S1 Table).

SP treatment of ectocervical explants resulted in a significant increase in the levels of all measured factors, except IL-1α and IL-10, after 2, 4, and 12 h of incubation with either SP50% (n = 11) or 25% (n = 7) (p<0.05) (Fig 1B and S1 Table). No significant difference in the magnitude of the induced cytokine response was observed between SP50% and SP25% at any time point. Of note, indomethacin treatment did not significantly affect cytokine increase associated with SP50% treatment, indicating that endogenous prostaglandin production in explants is not involved in the acute response to SP. In preliminary experiments, treatment of explants with lipopolysaccharides (LPS) recapitulated the increase in IL-6 and CXCL8 levels associated with the response to SP (n = 3, S3 Fig). Nevertheless, LPS-induced production of both proinflammatory cytokines was reduced by indomethacin treatment, suggesting that Toll-like receptor (TLR) 4 ligands present in semen may contribute to elicit an inflammatory response in the FGM, as reported in mice [20], although its genesis is likely to be multifactorial.

The same cytokines measured in ECM were also present in the SP pools used to treat explants (S1 Table). To address the contribution of seminal cytokines to the observed ECM cytokine increase associated with SP treatment, we functionally inactivated explants with paraformaldehyde (PFA). The effectiveness of explant inactivation was demonstrated by the absence of cytokine release during and after incubation with CM for all measured factors (CM-PFA) (S4A Fig). By comparing cytokine concentration values measured in ECM of SP-treated explants (SP) and the same values corrected for seminal cytokine carry-over (SP-PFA), we did not detect any significant difference between n-fold change values (median) for any measured factors, with the exception of a reduction of CCL5 (5.4 vs 4.2) and TGF-β1 (10.2 vs. 1.8) (n = 5, p = 0.062) (S4B Fig). Whereas the magnitude of carry-over appears negligible for CCL5, we excluded from our analysis the results on TGF-β1 ECM concentration as heavily affected by unspecific retention and release of seminal cytokine, likely from the agarose surrounding the tissue. These data indicate that the cytokine response elicited by SP originated within explants for most measured factors, despite the minor presence of residual seminal cytokines in ECM.

#### b) Gene expression in tissue explants

**Fig 2:**
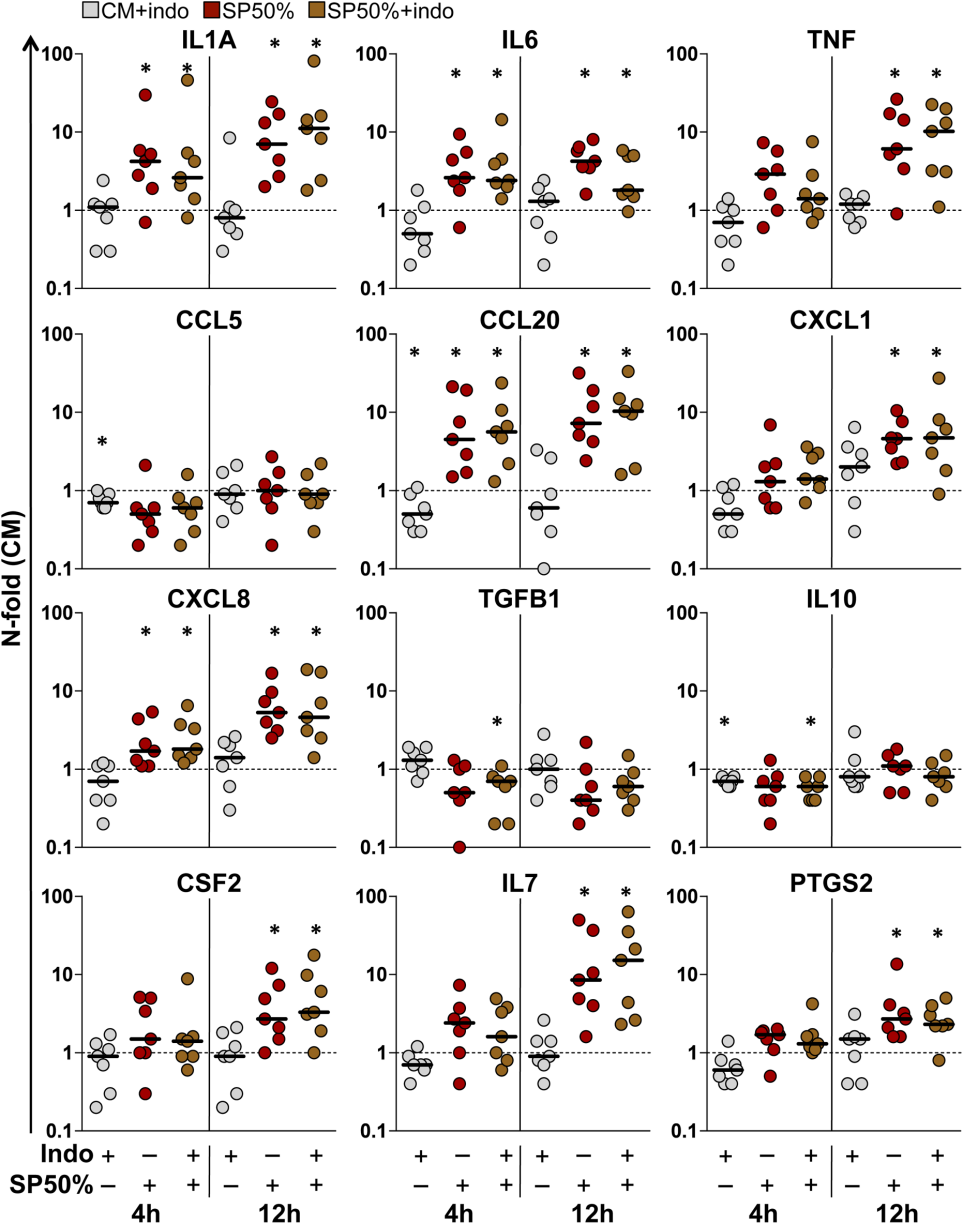
Gene expression in ectocervical tissue explants. N-fold change in gene expression measured in ectocervical explants incubated with indomethacin (indo) 10μM (gray), and seminal plasma (SP) 50% without (red) or with (brown) indomethacin for 4 or 12 h, compared to donor-matched explants incubated with culture medium (CM) (n = 7), followed by an additional 12 h-incubation with medium only. Bars indicate median values. Asterisks denote a significant difference with CM (Wilcoxon signed rank test, p<0.05).

The expression levels of the genes encoding the cytokines screened in explant culture supernatant were measured in explants harvested at the same of time of ECM (S1A Fig). The expression of *IL6*, *TNF*, *CCL20*, *CXCL1* and *CXCL8* was significantly upregulated in ectocervical explants treated with SP50% for 12 h, compared to untreated control explants (CM) (n = 7, p<0.05) (Fig 2 and S2 Table). Similar to protein levels, a significant increase in the levels of *IL6*, *CCL20* and *CXCL8* was already detectable after a 4 h-incubation. However, differences in the dynamics of upregulation between gene expression and respective ECM protein concentration were also observed for some factors. In particular, *IL1A* was significantly increased, whereas *CCL5* was unaffected by SP treatment for both treatment time lengths. After a 12 h-incubation with SP, we detected a significant increase in the expression of additional factors of interest, namely colony stimulating factor 2 (*CSF2*) and prostaglandin-endoperoxide synthase 2 (*PTGS2*), that were included in the analysis because of their implication in the response to semen *in vivo* [7,21], and *IL7*, a lymphotropic cytokine that was previously shown to promote HIV-1 replication in the FGM *ex vivo* [22] (Fig 2 and S2 Table). As for cytokine levels in ECM, indomethacin treatment did not significantly affect the basal expression levels of all measured factors, except for a reduction (median) of *CCL5* (0.7-fold), *CCL20* (0.5-fold), and *IL10* (0.7-fold) after a 4 h-incubation (n = 7, p<0.05) (Fig 2 and S2 Table). Importantly, indomethacin did not inhibit the increase in gene expression induced by SP treatment, confirming the observation that endogenous production of prostaglandins in explants does not contribute to the early mucosal response to SP.

### SP treatment is associated with increased leukocyte migration

**Fig 3:**
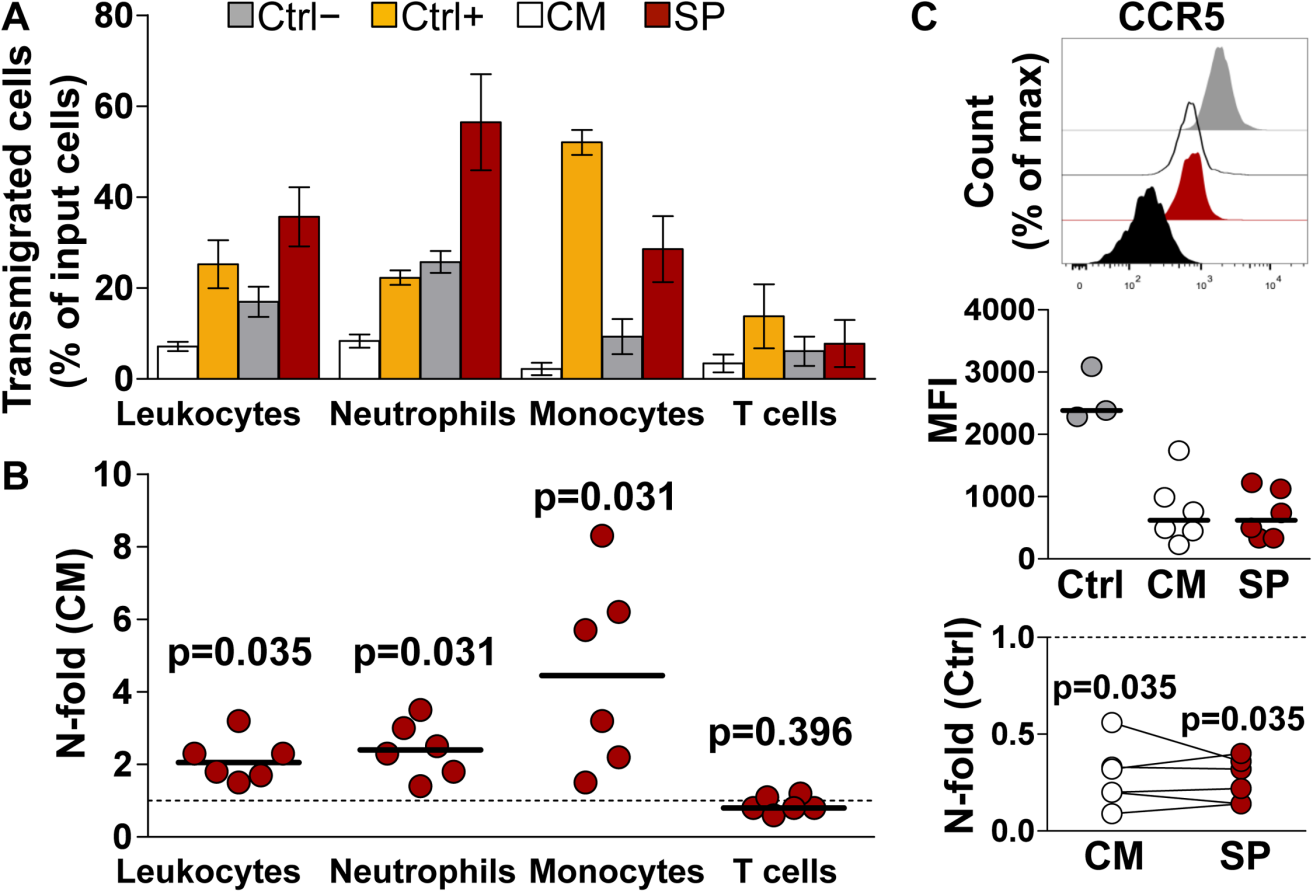
Transwell migration of peripheral blood leukocytes (PBL). Mononuclear cells and granulocytes were isolated from blood, pooled (i.e. PBL) and incubated in a transwell system for 2 h with explant conditioned medium (ECM) from donor-matched ectocervical explants incubated with culture medium (CM-ECM) or seminal plasma (SP-ECM). Transmigrated cells were immunophenotyped and enumerated by flow cytometry (see S5 Fig). **A)** Fraction of transmigrated cells out of total number of PBL loaded into a transwell insert for each analyzed cell population (input). PBL were incubated with CM-ECM (white), SP-ECM (red), and with medium only (grey) and medium supplemented with FBS 10% (yellow) as negative and positive controls respectively. Bars represent mean with s.e.m (n = 6). **B)** N-fold change in the fraction of transmigrated PBL incubated with SP-ECM compared to that of donor-matched CM-ECM. Bars indicate median values. p<0.05 denotes a significant difference with CM (Wilcoxon signed rank test). **C)** Expression levels of the chemokine receptor CCR5 on transmigrated monocytes. Top, peaks represent PBL untreated cultured (ctrl, gray), cultured with CM-ECM (CM, white), cultured with SP-ECM (SP, red), and unstained control (black) from one representative experiment. Middle, CCR5 mean fluorescence intensity (MFI). Bars indicate median values. Bottom, n-fold change in CCR5 MFI on PBL cultured with ECM compared to untreated cultured PBL (ctrl). Lines connect measurements obtained from donor-matched ECM. p<0.05 denotes a significant difference with ctrl (Wilcoxon signed rank test).

Local exposure to semen is associated with an influx of leukocytes into the FGM and rapid neutrophil egress from the mucosa into the vaginal lumen [9,21]. To investigate these events *in vitro*, we exposed peripheral blood leukocytes (PBL) from healthy donors to ECM for 2 h in order to assess its chemotactic power using a transwell assay. Input cells were obtained by reconstitution of the mononuclear cell and granulocyte fractions of blood. Neutrophils (CD16+CD66b+) were the predominant population among input cells, followed by T cells (CD16-CD3+) and monocytes (CD16-CD14+), reflecting the natural composition of circulating leukocytes (CD45+) (S5 Fig). Compared to ECM of untreated control explants (CM), incubation with ECM from SP-treated explants resulted in a median 2.0-fold increase in the number of transmigrated total leukocytes, and in particular a 2.4- and 4.4-fold increase in neutrophil and monocyte transmigration respectively (n = 6, p = 0.03) (Fig 3A & 3B). The relative contribution of the cellular and plasma fractions of semen to mucosal leukocyte recruitment in humans is not known. However, studies in animal models point at soluble factors in semen such as TGF-β1, prostaglandin-E, and TLR4 ligands as potential inflammatory mediators [7,20,23]. Although we did not investigate the role of those molecules, except for preliminary data on explant cytokine response to LPS (S3 Fig), we asked whether SP-mediated chemokine upregulation measured in ECM could explain the observed PBL migration. To this end, we measured the expression levels of the chemokine receptors CCR5, binding CCL5 among other β-chemokines, and CXCR1 and CXCR2, binding CXCL1 and CXCL8, on transmigrated cells as indirect evidence of cognate chemokine binding to the receptor. The mean fluorescence intensity (MFI) of CCR5 on transmigrated monocytes significantly decreased (median) following incubation with ECM from both CM- (0.26-fold) and SP-treated (0.27-fold) explants compared to untreated cultured PBL (n = 6, p = 0.035) (Fig 3C). There was no difference between n-fold change values of CCR5 MFI of the two treatments (p>0.999). These data suggest that increased local production of CCL5, as measured in SP-ECM, may account for semen-induced recruitment of monocytes to the FGM.

The expression of CXCR1 and CXCR2 on transmigrated granulocytes, identified among CD45+ cells based on their light-scattering properties (S5 Fig) did not decrease as we expected to. On the contrary, CXCR2 MFI on cells incubated with ECM from both CM- and SP-treated explants showed an increase compared to untreated cultured control cells, whereas CXCR1 levels were unchanged (S6 Fig). Of note, our analysis of transmigrated PBL was restricted to a 2 h-incubation with ECM, although the expression of chemokine receptors may vary in time, also under regulation of other factors than receptor ligands. Therefore, chemokine-neutralization experiments are required to substantiate our observations.

### SP is not toxic to ectocervical tissue explants

**Fig 4:**
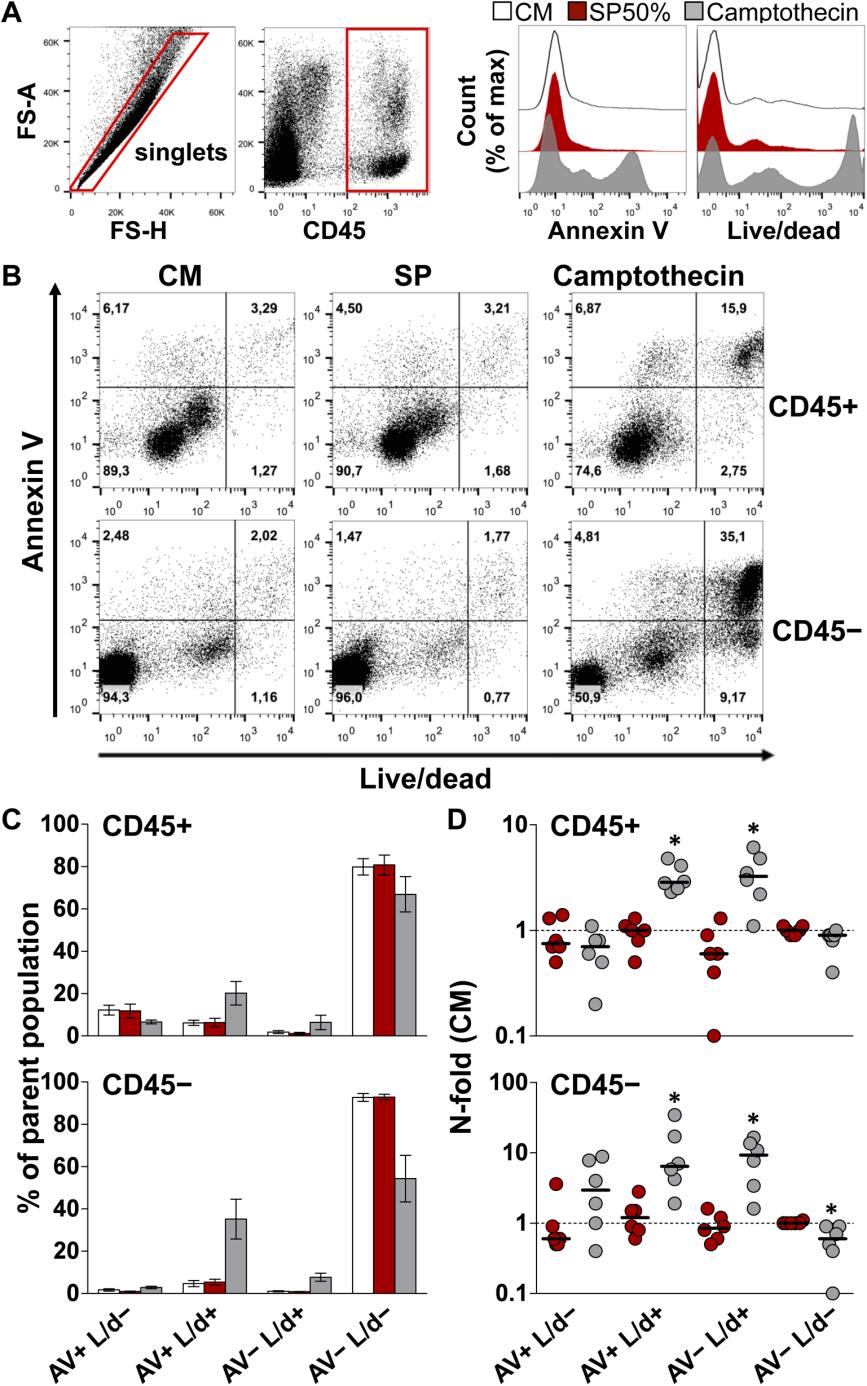
Viability of cells isolated from ectocervical tissue explants. Cells were isolated from ectocervical explants after incubation with culture medium (CM, white) or seminal plasma (SP, red) 50% for 12 h, followed by an additional 12 h-incubation with medium only. As a positive control, camptothecin 100 μM (grey) was used to induce apoptosis upon explant treatment for 24 h. Cells were stained with annexin V (AV) and an amine-reactive dye (Live/dead, L/d) and analyzed by flow cytometry. **A)** Representative dot plots of the gating strategy. Expression analysis of the two selected cell death markers was conducted on events phenotyped as immune (CD45+) and non-immune (CD45-) cells. Histograms depict the expression level of the cell death markers on singlets, which comprise both CD45+ and CD45- cells, isolated from donor-matched explants of one representative experiment. **B)** Dot plots of the fraction of cells expressing the two analyzed cell death markers among CD45+ (upper panel) and CD45- (lower panel) cells isolated from donor-matched explants of one representative experiment. **C)** Fraction of CD45+ (upper chart) and CD45- (lower chart) cells expressing different combinations of the two analyzed cell death markers. Bars represent mean with s.e.m (n = 6). **D)** N-fold change in the fraction of CD45+ (upper chart) and CD45- (lower chart) cells expressing different combinations of the two analyzed cell death markers from explants treated with SP (red) and camptothecin (grey) compared to untreated donor-matched explants (CM). Bars indicate median values. Asterisks denote a statistically significant difference with CM (Wilcoxon signed rank test, p<0.05).

A primary limitation of the experimental study of semen is its toxicity to isolated cells incubated with low concentrations of whole semen or SP (e.g., 10%) for 3 to 6 h [13,24,25]. We wanted to verify whether SP treatment affected cell viability in our system. Loss of membrane integrity was characterized by staining cells isolated from explants with an amine-reactive dye (Live/dead, L/d), as indicator of necrotic or late-apoptotic cells, and annexin V (AV), which binds to phosphatidylserine exposed on the outer plasma membrane early during apoptosis. Compared to donor-matched explants incubated with CM, a 12 h-treatment with SP50% did not affect the fraction of cells positive to either one or both cell death markers. This was demonstrated in both immune and non-immune cells, as defined by CD45 expression (Fig 4).

As expected, treatment with the proapoptotic drug camptothecin significantly increased the fraction of double positive (AV+ L/d+) and live/dead-single positive (AV- L/d+) populations, among both CD45+ and CD45- cells, with a significant decrease in the fraction of the double negative (AV- L/d-) population among CD45- cells (p<0.05) (Fig 4D). Also considering that the cell isolation procedure likely contributed to cell death, our analysis showed that the majority of cells retained within explants after a 24 h-culture in agarose were viable, with fractions (mean ± s.e.m., n = 6) of cells negative for both cell death markers in CM- vs. SP-treated explants of 79.8 ± 3.8% vs. 80.7 ± 4.6% of CD45+ cells, and 92.7 ± 1.8% and 92.8 ± 1.3% of CD45- cells (Fig 4C).

*In situ* detection of chromatin fragmentation revealed a few apoptotic cells that were located in the upper layers of the epithelium of both explants incubated with SP50% and CM for 12 h, and to a lesser extent in donor-matched uncultured explants (S7 Fig). Also, shedding of the upper epithelial layers was visible in cultured explants for both treatments. Although we could not establish a measurable indicator of toxicity using this method, the presence of apoptotic cells and epithelial disruption appeared to vary with tissue donors and manipulation of individual explants rather than being associated with SP treatment.

### SP enhances HIV-1_BaL_ replication in ectocervical tissue explants

**Fig 5:**
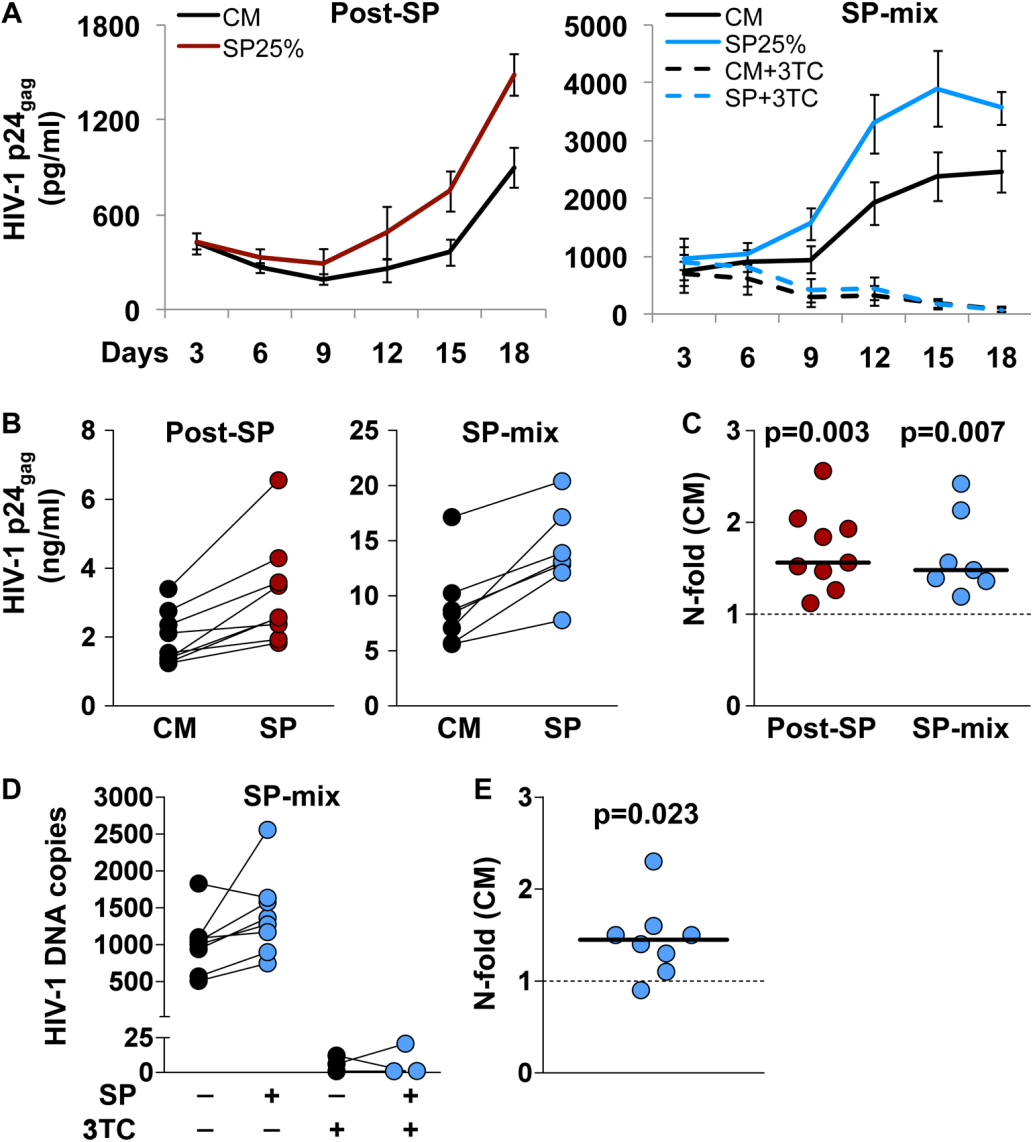
Infection of ectocervical tissue explants with HIV-1_BaL_. Infection of ectocervical explants with HIV-1_BaL_ was independently performed after seminal plasma (SP) 25% treatment (post-SP,red), or in the presence of SP25% (SP-mix, blue) (see S1B Fig). In selected experiments, explants infected in the presence of SP were treated with lamivudine (3TC) 10μM throughout culture time. **A)** Kinetics of HIV-1_BaL_ replication in explants treated with SP (colored line) or culture medium (CM) (black line). Virus replication was evaluated as p24_gag_ concentration in explant culture medium over 18 days. Represented are mean values with s.e.m. (n = 9 for post-SP; n = 8 for SP-mix; n = 3 for SP-mix+3TC). **B)** Cumulative p24_gag_ production over culture time. Lines connect measurements obtained from donor-matched explants. **C)** N-fold change in cumulative p24_gag_ production in SP-treated explants compared to donor-matched untreated explants. Bars indicate median values. p<0.05 denotes a significant difference with CM (Wilcoxon signed rank test). **D)** HIV-1 DNA quantification in explants infected with a mix of HIV and SP or CM, cultured in the presence or the absence of 3TC, and harvested at the end of culture (day18). HIV-1 DNA copy numbers were normalized to the amount of the single-copy gene *HBB*. Lines connect measurements obtained from donor-matched explants. **E)** N-fold change in HIV-1 DNA copy numbers in SP-treated explants compared to donor-matched untreated explants. The bar indicates median value. p<0.05 denotes a significant difference with CM (Wilcoxon signed rank test).

It has been speculated that the inflammatory nature of the response elicited by semen can affect the FGM susceptibility to HIV-1 [10]. To test this hypothesis, we infected ectocervical tissue explants with HIV-1 either following exposure to SP or in the presence of SP, as a model of HIV-1 transmission to the FGM (S1B Fig). Productive infection with the CCR5-tropic laboratory-adapted virus HIV-1_BaL_ was achieved for explants from all included donors (n = 17) regardless of the infection set-up, as evaluated by measuring the amount of HIV-1 core protein p24_gag_ released into culture supernatant over time (Fig 5A). Exposure of explants to SP25% for 4 h before infection (post-SP) resulted in a median cumulative p24_gag_ production of 2.5 ng/ml compared to 1.5 ng/ml for untreated explants (CM) (Fig 5B). Compared to donor-matched untreated explants, SP treatment resulted in a median 1.6-fold increase in cumulative p24_gag_ production (n = 9, p = 0.003) (Fig 5C). Similarly, infection of tissue explants with a mixture of HIV-1_BaL_ and SP25% for 12 h (SP-mix) resulted in a median cumulative p24_gag_ production of 12.9 ng/ml compared to 7.7 ng/ml for untreated explants (Fig 5B), that was equal to a median 1.5-fold increase as measured in donor-matched tissue explants (n = 8, p = 0.007) (Fig 5C).

To confirm the enhancing effect of SP treatment on HIV-1 replication we measured the amount of HIV-1 DNA in explants infected in the presence of SP (SP-mix) harvested at the end of culture (day 18). In agreement with the results on HIV-1 p24_gag_ levels, SP-treated explants showed a median number of HIV-1 DNA copies of 1319 compared to 1024 for untreated explants (CM) (Fig 5D), resulting in a median 1.4-fold increase as measured in donor-matched tissue explants (n = 8, p = 0.023) (Fig 5E). The data on HIV-1 p24_gag_ levels were consistent with HIV-1 DNA detection also for HIV-infected explants treated with the anti-retroviral drug lamivudine (3TC) throughout culture as a negative control (n = 3), showing a constant decline in HIV-1 replication kinetics associated with sporadic detection of HIV-1 DNA below 25 copies in explants harvested at the end of culture (day 18) (Fig 5A & 5D).

Of note, the use of progesterone-based drugs (n = 6), namely a levonorgestrel-containing IUD (n = 3) and per oral ulipristal acetate (n = 3), was associated with a lower SP-mediated increase in HIV-1 cumulative p24_gag_ production compared to explants from donors who did not use any hormonal drugs at the time of surgery (n = 11) (1.4 vs. 1.8 median n-fold, p = 0.056) (S8 Fig). Nevertheless, SP treatment significantly enhanced cumulative HIV-1 replication compared to donor-matched untreated explants in both groups (p<0.05). The effect of sex hormones on the local defenses of the FRT has been object of a number of investigations over the years, in particular with respect to the use of the injectable progestin medroxyprogesterone acetate in HIV endemic areas [26]. Although *in vivo* and *in vitro* experimental data point at a protective role of estrogen as opposed to progesterone [27,28], the mechanisms underlying hormonal regulation of HIV-1 transmission remain to be elucidated in humans. In addition, levonorgestrel and ulipristal acetate differ not only in the route of administration, but also in their affinity to the progesterone and glucocorticoid receptors among others. Therefore, the analysis of their effect on the local immune response in the lower FRT and on HIV-1 transmission therein requires a proper validation and study design.

Histologic analysis of HIV-infected ectocervical tissue explants harvested at the end of culture revealed the presence of HIV RNA in the stroma (S9 Fig), supporting the evidence that productive virus replication occurs within explants until the final days of culture, as already suggested by HIV-1 DNA results. In agreement with previous reports [29], the pluristratified epithelium characteristic of the lower FGM was not retained on ectocervical explants, as indicated by shedding of the upper epithelial layers already after 12 h of culture in agarose medium (S7 Fig). Nevertheless, the overall structure of the stroma, along with resident cells, was preserved upon infection. Morphology retention, although partial, accounts for a critical advantage offered by tissue explants over traditional monotypic and two-dimensional culture systems, which allows characterizing the early events regulating the founder pool of infected cells within their natural environment.

### Ectocervical tissue explants do not support transmitted/founder HIV-1 replication

**Fig 6:**
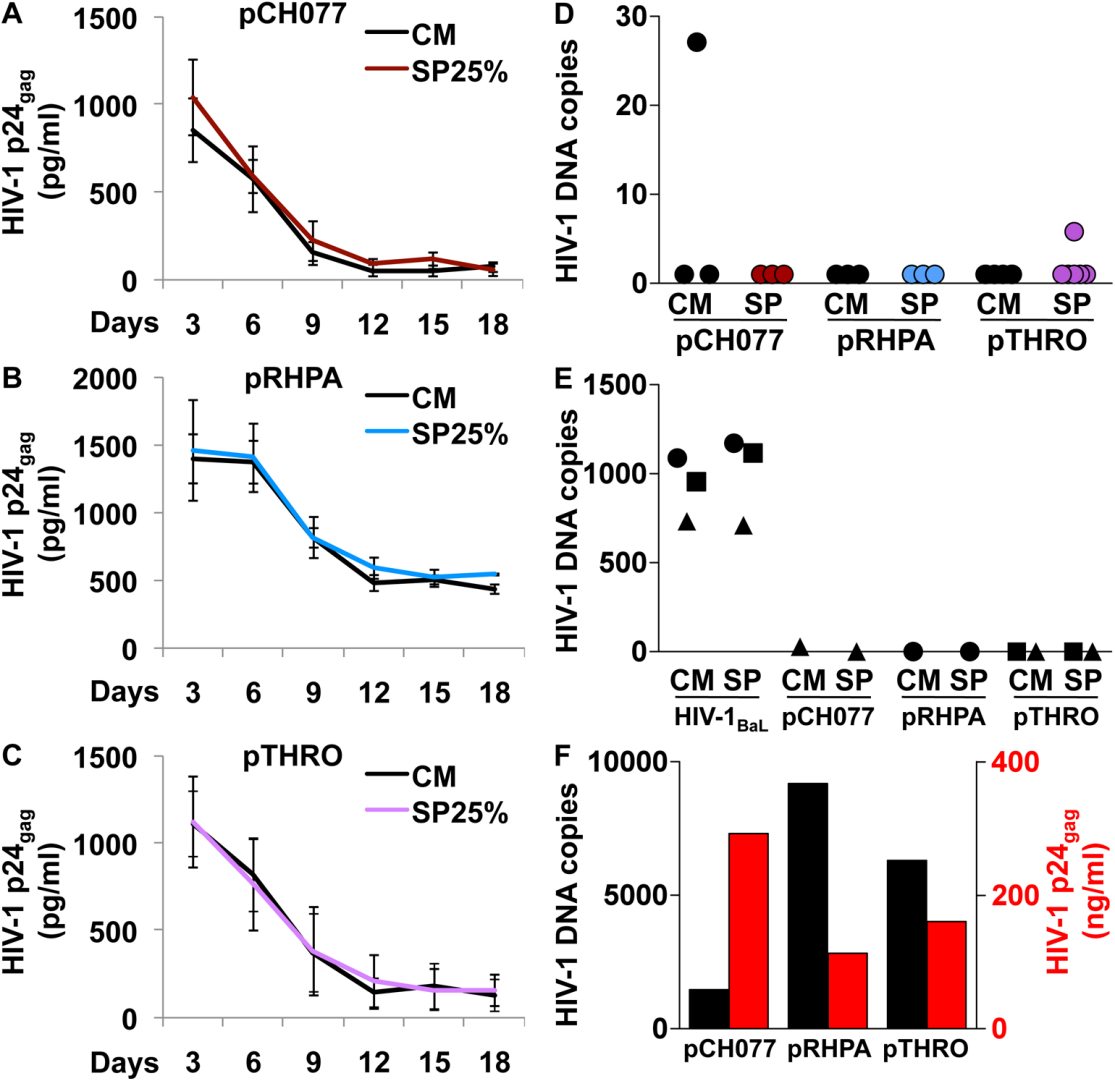
Infection of ectocervical tissue explants with transmitted/founder (T/F) HIV-1. Infection of ectocervical explants with pCH077.t/2627 (pCH077), pRHPA.c/2635 (pRHPA), and pTHRO.c/2626 (pTHRO) was performed after an initial treatment with seminal plasma (SP) 25% (see S1B Fig). **A-C)** Kinetics of virus replication in explants treated with SP (colored line) or culture medium (CM) (black line). Virus replication was evaluated as p24_gag_ concentration in explant culture medium over 18 days. Represented are mean values with s.e.m. (n = 3 for pCH077; n = 3 for pRHPA; n = 4 for pTHRO). **D-E)** HIV-1 DNA copy numbers in infected explants harvested at the end of culture (day18). Values were normalized to the amount of the single-copy gene *HBB*. **E)** Donor-matched explants were independently infected with HIV-1_BaL_ and at least one T/F virus (n = 3). Donor-matched explants are indicated with the same symbol. **F)** Exogenously activated PBMCs were infected with T/F HIV-1 produced in 293T cells. HIV-1 replication was evaluated as p24_gag_ concentration in culture supernatant (red) and HIV-1 DNA copy numbers (black) in cells harvested at the end of culture (day 8–10 post-infection) (n = 1).

To further validate our findings using viruses representative of mucosally acquired variants, we tested full length infectious molecular clones (IMCs) of viruses derived from circulating virions or infected cells of patients with acute HIV-1 clade B infection, also known as transmitted/founder (T/F) HIV-1 [30,31]. In particular, we selected the viruses pCH077.t/2627 (male host), pRHPA.c/2635 (female), and pTHRO.c/2626 (male) based on the limited data available on ectocervical tissue explant infection with T/F HIV-1 [32,33]. As we expected T/F HIV-1 to replicate at lower levels than HIV-1_BaL_, we decided to infect explants after SP treatment (post-SP) to avoid diluting the virus inoculum and maximize the time of infection (18 h). Nevertheless, we did not obtain a productive infection with any of the three selected T/F HIV-1, regardless of SP treatment, as showed by p24_gag_ levels in explant culture supernatant measured over time (Fig 6A–6C). Of note pRHPA.c/2635, the only virus isolated from a female host among those tested, exhibited a distinctive replication kinetics reaching a steady p24_gag_ production of about 500pg/ml during the last six days of culture (Fig 6B). The other two tested T/F HIV-1 generally showed lower p24_gag_ levels and undetectable levels at the end of culture in 1 out of 3, and 2 out 4 experiments for pCH077.t/2627 and pTHRO.c/2626 respectively (Fig 6A & 6C). HIV-1 DNA was detected in low copy numbers, comparable to those occasionally observed for HIV-1_BaL_-infected explants treated with 3TC (Fig 5D), in 1 out of 3 (CM only), and 1 out 4 (SP only) experiments for pCH077.t/2627 and pTHRO.c/2626 respectively, whereas HIV-1 DNA was undetectable in 3 out 3 experiments for pRHPA.c/2635 (Fig 6D). The complete absence of HIV-1 DNA in explants infected with pRHPA.c/2635 seems in contrast with p24_gag_ levels in culture medium of the same explants, although we cannot exclude that infected cells migrated from the explants into gelatin sponges. In addition, the inoculum of pRHPA.c/2635 was higher than the other two T/F HIV-1, thus possibly resulting in higher absorption and unspecific release of virus. In selected experiments (n = 3), we infected donor-matched tissue explants with HIV-1_BaL_ in parallel with one or more T/F HIV-1. Tissue explants from all three included tissue donors were susceptible to HIV-1_BaL_ but none of the T/F viruses, as evaluated by HIV-1 DNA quantification in explants harvested at the end of culture (Fig 6E). In addition, T/F viruses produced in 293T cells productively infected exogenously activated PBMCs as verified by p24_gag_ levels in culture supernatant and HIV-1 DNA copy numbers in cells harvested at the end of culture (day 8–10 post-infection) (Fig 6F).

## Discussion

This is the first work to attempt to extensively reproduce the immunoregulatory effect of SP on the FGM as described in humans, and combine it with HIV-1 infection *ex vivo* using human mucosal tissue from the lower FGT. Compared to previous studies employing human tissue explants to study specific aspects of semen-mediated inflammation, the goal of the present work is, by providing accurate tissue-donor and specimen inclusion criteria as well as multiple analytical readouts, to develop an experimental platform open to modifications for the study of the early molecular events regulating HIV-1 transmission to the FGM.

To the best of our knowledge, data on *in vivo* cytokine production in the human FRT following exposure to semen are limited to one recent study, which revealed an overall lack of change in the levels of most of the cytokines analyzed here, except for a reduction in CXCL8 and increase in TGF-β1, in cervicovaginal lavage collected 2 to 6 h after unprotected sex [34]. In mice, semen affects cytokine production at distal sites from the uterus [35], indicating that the bulk of genital secretions collected in the vagina, in addition to the dilution factor introduced with the collection method, may not accurately reflect local changes occurring at the site of semen exposure. *In vitro* experiments revealed that both epithelial and stromal cells isolated from the upper and lower FRT can produce and upregulate in response to SP the proinflammatory cytokines and chemokines measured here [13,36,37]. Also in agreement with our findings, enhanced production of CXCL8 and no change in IL-10 levels were previously reported in SP-treated human ectocervical tissue explants [38]. The levels of cytokine gene expression in explants generally recapitulated those measured in ECM, showing an induction of proinflammatory cytokines upon SP treatment. Our results are in agreement with those of Sharkey et al., who compared mRNA levels in ectocervical biopsies collected from healthy fertile women before and after unprotected vaginal coitus [21]. In their study, mucosal exposure to semen was associated with enhanced transcription of *IL1A*, *IL6*, *CXCL8*, and *CSF2*. In the same samples, *PTGS2*, *TNFA*, *CCL20*, and *CXCL1* were also upregulated by semen, although not significantly. Increased levels of both *PTGS2* and cyclooxygenase 2 were previously reported in SP-treated human ectocervical tissue explants [39], confirming our findings.

In contrast to early artificial fertilization studies implicating whole semen as the mediator of leukocyte recruitment to the FGM [8,9], our data indicate that SP alone can induce such response. As observed in those same reports and other studies in mammals [7], neutrophils and monocytes are the predominant migrating populations early after mucosal exposure to semen. Tissue infiltration by these cell types is indeed characteristic of acute inflammatory responses, and their interplay is key to the progress and eventual resolution of inflammation [40,41]. ECM from both CM- and SP-treated explants attracted a modest fraction of T cells, suggesting that their recruitment to the FGM may occur later on during the mucosal response to semen. This may be due to antigen presentation requirement in secondary lymphoid organs, as observed for post-coital mucosal infiltration of T cells specific to male antigens in mice [42,43]. Phenotypic analysis of transmigrated cells upon incubation with ECM, together with chemokine neutralization experiments, may provide useful information on the molecular mechanisms regulating immune cell recruitment to the FGM, as shown by CCR5 reduction on transmigrated monocytes in our system. In addition, phenotypic and functional analysis upon incubation with SP-ECM of cellular subsets of interest, isolated either from blood or the FRT, may contribute to the study of cell maturation and differentiation processes towards a local tolerogenic response mediated by SP, as observed for the T regulatory response in mice [43,44], as well as in human dendritic cells *in vitro* [45].

An important aspect of our model is that SP treatment did not affect cell viability as measured on individual cells isolated from explants after 24 h of culture in agarose. This sets an important difference with other *in vitro* systems based on primary cells or cell lines for the study of SP [13,24,25]. In those settings incubation with low dilutions of SP for short time lengths was associated with elevated toxicity, which is enhanced by the presence of serum [46]. In the present work we used charcoal stripped FBS at the final concentration of 2.5% to prepare agarose medium, and serum-free medium to dilute SP, treat explants as a control (CM) and incubate explants for 12 h after SP or CM treatment. Although the nature and low concentration of FBS could have contributed to limit cytotoxicity, the structural and functional integrity of tissue explants, even if partially preserved, may explain the absence of cell death observed upon treatment with highly concentrated SP (50%) for a relatively long time (12 h). This aspect offers a significant advantage over classical *in vitro* systems which results rely on possibly over-diluted semen or SP.

Our results suggest that the acute mucosal response to SP creates a favorable environment for virus replication early during transmission to the FRT, and are in line with reports implicating SP as an enhancer of HIV-1 infection [5,10]. Of note, we challenged explants with HIV-1_BaL_ after SP treatment at first (post-SP). This experimental setting provided a longer period for infection than the incubation with SP, 18 and 4 h respectively, in order to maximize the probability of obtaining a productive infection with the selected virus inoculum. In addition, we wanted to primarily address the effect of SP-induced mucosal changes on HIV-1 replication, avoiding any physical interactions between virions and seminal components, as a result of mixing SP and the virus stock, that could have eventually enhanced the infection [24,47]. Nevertheless, with this experimental setting we cannot exclude the presence during HIV-1 infection of seminal factors retained on the explant surface or within the surrounding agarose upon SP wash-out, as observed for seminal TGF-β1. However, to more closely mimic the biology of HIV-1 sexual transmission to the FGM, we also performed infection with a mixture of HIV-1_BaL_ and SP (SP-mix). In order not to extend explant exposure to SP over 12 h, as originally designed for cytokine experiments, and maximize the probability of obtaining a productive infection at the same time, we doubled the HIV-1_BaL_ inoculum used for the previous infection setting (post-SP) due to the shorter incubation time with the virus. This technical difference in the experimental set-up may explain the higher levels of HIV-1 replication achieved with HIV-SP mixing than SP treatment prior to infection, although a biological effect linked to the incubation and direct interaction between virus and seminal factors could have played a role as well [24,47]. As a future perspective, we would like to interfere with candidate seminal factors that signal to the mucosa, such as prostaglandins and TLR4 ligands [17,20,48], in order to harness the induced cytokine response and potentially modulate its effect on HIV-1 replication.

As opposed to infection with HIV-1_BaL_, the use of T/F HIV-1 full length IMCs did not result in detectable infection and replication in ectocervical tissue explants. These results evidence differences between HIV-1 isolates, as well as the experimental systems used to test them. In fact, most of the data available on T/F viruses have been produced using cell lines or exogenously activated primary cells [49,50]. Based on the limited data available to date, there is no robust experimental evidence that the T/F IMCs tested in our study can establish a productive infection in mucosal tissues as evaluated in explants from the FRT [32,33]. Nevertheless, those same studies failed to show any difference in transmission efficiency between T/F viruses and HIV-1_BaL_ among other reference chronic viruses [32,33], as also suggested by some *in vitro* studies, although the topic remains debated [51]. On the other hand, we do not exclude the possibility that T/F HIV-infected cells might have exited explants and accumulated into gelatin sponges thus explaining the sustained production of p24_gag_ observed in our system for pRHPA.c/2635-infected explants at the end of culture. Vaginal myeloid dendritic cells were shown to preferentially uptake T/F HIV-1 to trans-infect lymphocytes *in vitro* upon migration from human vaginal tissue inoculated with full length IMCs *ex vivo* [52].

Further analysis of the local cytokine milieu, as well as CD4 and CCR5 levels on virus target cells isolated from explants, may help to rule out the nature and the cause of the observed T/F HIV-1 replication kinetics. Explant activation might be required to boost the replication of less infectious HIV-1 variants, as observed for primary isolates *ex vivo* [53], as well as in women with pre-existing genital inflammation *in vivo* [54]. Finally, the comparison with primary isolates from acutely infected individuals may also reveal important differences in the biology of infection between isolates and molecular clones in a more complex system than isolated cells such as tissue explants.

One important limitation of our transmission model is that it does not accurately mimic the natural route and dynamics of virus entry into the mucosa due to the lack of explant polarization. However, it is a valid tool for studying the founder pool of HIV-1 infected cells, which fate is believed to ultimately determine the outcome of a transmission event [55]. Based on our results, it appears that the fraction of tissue-resident HIV-1 target cells present within explants are sufficient to support and modulate the magnitude of virus replication in response to SP, at least in the early stage of transmission. This hypothesis is supported by *in vivo* studies conducted in non-human primates, in which CD4+ T cell infiltrates became apparent only at day 4 post-infection with SIV, or later on during infection [56,57]. Of importance, the presence of infected cells within explants was confirmed by HIV-1 DNA quantification and *in situ* HIV RNA hybridization at the end of culture (day 18 post-infection), suggesting that HIV target cells residing within the FGM are either long-lived or undergo local replication. Based on previous studies of human cervicovaginal explants infected with HIV-1 and treated with the following factors, we speculate that SP promotes viral replication by upregulating proinflammatory cytokines, such as IL-6 [58] and CXCL8 [59], as well as lymphotropic growth factors such as IL-7 [22]. These factors can directly upregulate HIV-1 gene transcription, and increase the activation and life-span of HIV-infected and bystander target cells. However, the recruitment of immune cells to the transmission site is likely required for amplification and systemic spread of the infection *in vivo*. For instance, in our system SP upregulated the production of CCL20, a chemokine that was previously shown to be involved in promoting HIV-1 infection of the FGM through recruitment of Langerhans cells *in vitro* [60], and plasmacytoid dendritic cells in non-human primates already at day 1 post-infection with SIV [56,57]. Moreover, T helper 17 cells expressing the CCL20-binding receptor CCR6 were identified as preferential targets of SIV during vaginal transmission [61].

Of importance, intravaginal challenge with SIV is usually performed in the absence of SP, and this may affect the nature of the local response in the FGM as well as the composition of infiltrating cells. As mentioned above, mucosal exposure to semen in mice resulted in the establishment of local tolerance to paternal antigens in the FRT [43]. The response triggered by the combination of HIV-1 infection and SP treatment may have peculiar features distinct from the two separate treatments. This point remains unaddressed in the present study, as leukocyte transmigration experiments were performed using ECM from explants treated with SP from HIV-uninfected individuals or CM in the absence of HIV-1, in order to specifically investigate the mucosal response to SP. Therefore it is challenging to extrapolate implications for HIV-1 transmission from our results on ECM cytokines and cell transmigration in response to ECM. The use of SP from HIV-infected individuals to treat explants and related ECM, would be more relevant to this end. It is indeed well know that HIV-1 infection, as well as the stage of infection, can significantly affect the immunologic and microbiologic profiles of semen, with potential implications for virus transmission [62–64]. Cell-associated HIV is also an important source of transmitted virus [65–67] that was not addressed in the present study and may be object of future investigations.

The evidence that HIV-1 transmission to the FGM is inefficient [1] may not reflect the likelihood of a locally productive infection being established, the propagation of which would require the maintenance of an inflammatory environment as modeled in our system, but rather the ability of the mucosa to rapidly counteract semen-induced inflammation, among other inflammatory conditions. The response to semen is transient *in vivo* and may resolve passively once semen is washed out, or through activation of endogenous or seminal factors at a later stage [7]. For instance, TGF-β is highly concentrated in SP as inactive precursor and is reportedly activated in the FGM by numerous mechanisms [36]. The magnitude of the TGF-β1 response, as well as that of other seminal and mucosal factors *in vivo*, may be affected by modification of the tissue microenvironment by infiltrating leukocytes. Therefore, our system cannot completely reproduce the events following the acute response to semen and particularly the local tolerogenic or low inflammatory environment observed in animal models days post-exposure [43], and in women experiencing frequent unprotected sexual intercourse as sex workers [68,69].

In conclusion, the experimental system presented here reproduces the early events surrounding the interaction between SP and the FGM. Our results suggest that semen and the FGM do not act as a passive carrier and recipient of HIV-1, and understanding their crosstalk may reveal important clues about local susceptibility to infections, as well as other inflammatory disorders of the FRT, such as infertility and cancer. As evidenced by others [2,70], our study supports the notion that including semen or its components in the design of experimental and preclinical models of HIV-1 transmission may increase the relevance of their findings accelerating the discovery of new prevention measures.

## Materials and methods

### Ethics statement

Approval for the collection of human uterine samples was obtained from the Regional Ethical Review Board of Stockholm. Written informed consent was obtained from all tissue donors. Medical history information was collected from tissue donors via a questionnaire at the time of surgery.

### Semen collection

Semen was obtained via masturbation after ≥48 h of abstinence from 15 donors (aged 18 to 65 years) recruited at the Venhälsan Clinic of the Södersjukhuset, Stockholm. Exclusion criteria were: HIV seropositivity, clinical symptoms of sexually transmitted disease within 3 months prior to donation, systemic immunosuppressive therapy, and infertility (if known). The HIV test was conducted using an Alere Determine HIV-1/2 antibody test (Alere, Waltham MA, USA) at the Venhälsan Clinic.

Semen specimens were collected in a sterile container, allowed to liquefy at room temperature (RT) and processed within 60 min from collection. Specimens were supplemented with penicillin-streptomycin 100U/ml, gentamicin 50μg/ml, Fungizone 2.5μg/ml (all from ThermoFisher Scientific, Waltham MA, USA), and bovine serum albumin (BSA) 0.1% (Sigma-Aldrich, St. Louis MO, USA). SP was separated from cells by centrifugation at 700×*g* for 15 min, clarified by centrifugation at 17000×*g* for 5 min in a new tube, aliquoted and stored at -80°C. For treatment of ectocervical tissue explants, aliquots of SP from five randomly selected donors were thawed and pooled in equal proportion before each experiment. Cytokine concentrations in the SP pools used for cytokine measurements in explants and related ECM are reported in S1 Table and S4 Fig.

### Ectocervical tissue collection

Uterine specimens were obtained from patients undergoing hysterectomy for benign conditions, such as heavy/irregular menstrual bleeding or benign myoma, at the St. Göran Hospital, Stockholm. Exclusion criteria were: clinical symptoms of sexually transmitted disease within 3 months prior to surgery, systemic immunosuppressive therapy, and human papilloma virus (HPV) positivity. HPV genotyping was conducted on cervical swabs using a PapilloCheck HPV test (Greiner Bio-One GmbH, Kremsmünster, Austria) at the accredited microbiology laboratory of the Karolinska University Hospital.

Culture medium (CM) was prepared by supplementing RPMI 1640 with gentamicin 50μg/ml, Fungizone 2.5μg/ml, non-essential amino acids, and sodium pyruvate 1mM (all from ThermoFisher Scientific). A 4-9cm^2^ sample of mucosa was dissected from the uterine ectocervix by a pathologist immediately after hysterectomy and maintained in CM at 4°C for transportation. Ectocervical tissue was processed within 6–8 h from surgery. The mucosa was dissected into tissue blocks (i.e., explants) of approximately 8 mm^3^. One explant was snap-frozen immediately after dissection and cryopreserved at -80°C for *in situ* terminal deoxynucleotidyl transferase dUTP nick end labeling (TUNEL) assay (S1A Fig).

### Treatment of ectocervical tissue explants

Dissected ectocervical explants were placed at the bottom of a 24-well plate (1 explant per well) with the epithelial side facing upwards (S1A Fig). Due to the limited amount of tissue available, only one technical replicate was performed for each experimental condition. Agarose gel 4% (ThermoFisher Scientific) was melted at 70°C for 10 min and equilibrated at 37°C for 2 min. Agarose was mixed 1:1 with CM supplemented with charcoal stripped FBS 5% (ThermoFisher Scientific) pre-warmed at 37°C. A volume of 300–500μl of agarose medium was used to embed ectocervical tissue explants in the 24-well plate to cover all cut surfaces, leaving the epithelial side exposed to the air. Agarose was allowed to solidify at RT for 2 min, before adding 400μl of serum-free CM into the well. The explants were incubated at 37°C, CO_2_ 5%, and humidity 95% for 1 h. The supernatant was removed, and the explants incubated with serum-free CM or SP. Pooled SP was diluted 1:1 (50%, v/v) or 1:3 (25%) in serum-free CM. Explants were treated by adding 400μl of CM (untreated control) or SP to the well and incubated at 37°C, CO_2_ 5%, and humidity 95% for 2, 4, or 12 h. The supernatant was then removed, and the explants were harvested and snap-frozen for *in situ* TUNEL, or washed and incubated with 500μl of fresh serum-free CM at 37°C (S1A Fig). After 12 h, the supernatant (i.e., ECM) was collected, clarified by centrifugation at 17000×*g* for 1 min, and stored at -80°C. Explants were harvested and soaked in RNA*later* RNA Stabilization Reagent (Qiagen, Hilden, Germany) before storage at -80°C.

To evaluate SP cytokine carry-over, after dissection explants were soaked in a solution of phosphate-buffered saline (PBS) and PFA 4% (Sigma-Aldrich) at RT for 2 h and subsequently at 4°C for 12h. Donor-matched fresh and PFA-inactivated explants were treated with CM and SP50% for 12 h, as described above. The CM and SP solutions used to treat explants were harvested before washing the explants, clarified by centrifugation at 17000×*g* for 1 min, and stored at -80°C along with a sample of fresh CM collected immediately after washing. Explants were incubated with 500μl of fresh CM at 37°C for 12 h. Thereafter ECM was collected, clarified by centrifugation at 17000×*g* for 1 min, and stored at -80°C.

The cyclooxygenase-inhibitor indomethacin (Sigma-Aldrich) was reconstituted in absolute ethanol at the concentration of 10mM and stored at -20°C for maximum 2 months. For indomethacin treatment, indomethacin was added to CM and SP50% at the concentration of 10μM, and 30 μM in selected experiments, and to fresh CM for the following 12h-incubation. In selected experiments, explants were also treated with lipopolysaccharides (LPS) from *Escherichia coli* O11:B4 (Sigma-Aldrich) at 2μg/ml for 12 h, in the presence or the absence of indomethacin 10μM and 30μM.

To evaluate toxicity of SP treatment, donor-matched explants were treated with CM and SP50% for 12 h and subsequently incubated with fresh CM for 12 h, as described above. As a positive control, explants were treated with a solution of medium and the topoisomerase-inhibitor camptothecin (Sigma-Aldrich) at the concentration of 100μM for 24 h to induce apoptosis. At the end of culture, explants were harvested and immediately processed for cell isolation. A total of 9 explants (3 explants per well) were used for each experimental condition.

### Bead-array immunoassay

Cytokine concentration in ECM was measured using a multiplex bead-array immunoassay, developed as previously described [71]. All monoclonal capture antibodies, biotinylated polyclonal detection antibodies, and human recombinant cytokines were purchased from R&D Systems (Minneapolis MN, USA), except for IL-10 capture and detection antibodies that were purchased from BioLegend (San Diego CA, USA). Individual magnetic carboxylated bead sets (Luminex, Austin TX, USA) were coupled to the capture antibodies according to the manufacturer’s recommendations. Human recombinant cytokines were resuspended at concentrations ranging from 5 to 10ng/ml, and diluted serially 1:3 to generate standard curves. All assay procedures were performed in a buffer containing PBS supplemented with BSA 0.1%. The assay was run using 2000 beads per bead set in a total volume of 50μL per well. Samples of ECM were run in duplicates at 2 dilutions 1:1 and 1:9. 50μl of sample were added to the bead mixture and incubated overnight at 4°C in a Bio-Plex Pro flat bottom 96-well plate (Bio-Rad). Plates were washed twice with PBS containing Tween-20 0.05%. The beads were incubated with the detection antibody mixture for 60 min at RT. Biotinylated detection antibodies were used at twice the concentrations for a classic enzyme-linked immunosorbent assay recommended by the manufacturer. Plates were washed twice, and the beads incubated with a solution containing phycoerythrin-conjugated streptavidin 6μg/ml (ThermoFisher Scientific) for 30 min at RT. Beads were acquired with a Bio-Plex100 system (Bio-Rad). The median fluorescence intensity of a minimum of 100 beads per each bead set was recorded in each sample, and analyzed with the Bio-Plex Manager software (Bio-Rad) using a 5P regression algorithm. Concentration values were normalized to explant weight. Concentration values that were below the lower limit of quantification (LLOQ) were reported as the midpoint between zero and the LLOQ for statistical analysis. The LLOQ (pg/ml) was 2.0 for IL-1α, 4.6 for IL-6, 11.0 for TNF, 11.0 for CCL5, 20.6 for CCL20, 2.0 for CXCL1, 3.6 for CXCL8, 6.8 for TGF-β1, and 2.0 for IL-10.

### RNA extraction and quantitative real-time PCR

Ectocervical explants preserved in RNA*later* were weighed, washed with PBS, and processed to purify RNA using the RNeasy Mini Kit (Qiagen) according to the manufacturer’s instructions. Eluted RNA was treated with DNase for 30 min at 37°C using a TURBO DNA-free kit (ThermoFisher Scientific). RNA was reversed transcribed into first-strand cDNA using a SuperScriptII reverse transcriptase kit, random hexamers 2.5μM, and dNTP mix 0.5mM (all from ThermoFisher Scientific).

The sequences of the primers used for quantitative real-time PCR are listed in S3 Table. Some primer pairs were designed using the software Primer-BLAST [72,73]. All primers were purchased from ThermoFisher Scientific. Serial dilutions of cDNA were used to evaluate the amplification efficiency of each pair of primers as determined by regression analysis of amplicon abundance vs. threshold cycle (Ct). All used primers had efficiencies within 90 and 110%. Primer specificity was evaluated from the dissociation curve profile of PCR products. PCR amplification was performed in a 20μl-reaction containing HOT FIREPol EvaGreen qPCR Supermix (Solis BioDyne, Tartu, Estonia), forward and reverse primers 0.25μM each, and 3μl of cDNA, using an ABI Prism 7500 real-time PCR system (Applied Biosystems, Foster City CA, USA). The thermal cycler profile was as follows: 95°C for 12 min, 40 cycles of 95°C for 15 sec, 60°C for 30 sec, and 72°C for 1 min. PCR products were analyzed by dissociation curve profile. Samples were run in duplicate.

The relative quantity (RQ) of genes of interest (GOI) was calculated using the equation 2^ ^-ΔCt^, where ΔCt is the difference between the Ct of the GOI and the Ct of an endogenous reference gene. N-fold values were calculated using the equation 2^ ^-ΔΔCt^, where ΔΔCt is the ratio between the GOI RQ in SP and/or indomethacin-treated explants, and the GOI RQ in donor-matched untreated explants (CM). The genes encoding for glyceraldehyde 3-phosphate dehydrogenase (*GAPDH*), beta-actin (*ACTB*), and ubiquitin C (*UBC*) were used as endogenous references. In each independent experiment, GOI RQ and n-fold values were calculated individually for the 3 endogenous reference genes, and individual n-fold values were averaged to perform statistics.

### Blood collection and transmigration assay

Peripheral blood was collected from healthy volunteers at the Karolinska University Hospital. Peripheral blood mononuclear cells (PBMCs) were isolated by density gradient centrifugation at 400×*g* for 20 min using Ficoll-Hypaque (GE Healthcare, Wilmington MA, USA). Granulocytes were recovered from the resulting pellet upon resuspension in a solution containing EDTA 0.1mM, NH_4_Cl 180.0mM, and KCO_3_ 10.0mM to lyse erythrocytes. The PBMC and granulocyte fractions were counted, resuspended at 1×10^6^ cells/ml in medium, and mixed 1:1 (i.e. PBL). A total of 4×10^5^ PBL (400μl) were added into a 24-well plate insert with a 5μm-pore filter (Millipore, Billerica MA, USA) and incubated with 800μl of ECM from donor-matched CM- or SP-treated explants diluted 1:3 in medium added into the well, for 2 h at 37°C, CO_2_ 5%, and humidity 95%. PBL were incubated with medium only or medium supplemented with FBS 10% as a negative and positive control respectively. Due to the limited volume of ECM available, only one technical replicate was performed for each experimental condition. The cell suspension in the wells was harvested, and 50μl of CountBright beads (ThermoFisher Scientific) was added to each sample. Cells were washed with PBS EDTA 1mM, and stained with the following antibodies: CD45-V450 (HI30), CD3-V500 (UCHT1), CD14-FITC (MφP9), CD66b-PE (G10F5), CD195 (CCR5)-APC (2D7), CD16-APC-Cy7 (3G8), CD181 (CXCR1)-PE (5A12), and CD182 (CXCR2)-APC (6C6) (all from BD Biosciences, San Jose, CA, USA). For each sample a minimum of 1x10^4^ events were recorded in the gate corresponding to the beads using a Gallios analyzer (Beckman Coulter, Brea CA, USA) (S5 Fig). Data were analyzed using FlowJo v10.0.7 (FlowJo, Ashland OR, USA). To compare the number of acquired events between samples within the same experiment, the number of cells was normalized to the number of beads.

### Cell isolation and flow cytometry

Ectocervical explants were digested using a solution of Hank’s balanced salt solution with calcium and magnesium (ThermoFisher Scientific), DNase I grade II at 100μg/ml and Liberase Dispase Low Research Grade at 16μg/ml (0.08 Wünsch units/ml) at 37°C for 1 h rocking at 1500 revolution per minutes using a thermomixer (1ml for 9 explants in a 1.5ml microtube). Both enzymes were purchased from Roche Diagnostics (Mannheim, Germany) and reconstituted in distilled cell culture grade water at the concentration of 1mg/ml for storage at -20°C. After tissue digestion, the supernatant was filtered on a 100μm-cell strainer (BD Biosciences) and explants were mechanically disrupted using a pestle, rinsed with PBS EDTA 1mM and the supernatant transferred onto the filter. Cells were washed twice with PBS EDTA 1mM and spun at 250x*g* for 5 min. Cells were stained separately with the LIVE/DEAD far red dead cell staining kit (ThermoFisher Scientific), CD45-Pacific Blue (HI30) (BioLegend), and Annexin V-PE (BD Biosciences) according to the manufacturers’ instructions. Events were acquired using a CyAn ADP analyzer (Beckman Coulter) and data were analyzed using FlowJo v10.0.7 (FlowJo).

### *In situ* TUNEL

Cryopreserved samples of ectocervical tissue explants were mounted in optimal cutting temperature (OCT) embedding medium (Histolab Products, Askim, Sweden) and cut into 8μm-thick sections. Sections were fixed in PFA 2% (Sigma-Aldrich) at RT for 10 min, and processed to visualize chromatin fragmentation using the terminal deoxynucleotidyl transferase dUTP nick end labeling (TUNEL) kit TACS-XL according to manufacturer's instructions (Trevigen Bio-Techne). Briefly, sections were treated with proteinase K 1:60 for 15 min, hydrogen peroxide 3% for 10 min, and incubated with a mixture of terminal deoxynucleotidyl transferase (TdT) and brominated nucleotides (BrdU) for 60 min at 37°C. The TdT enzyme was omitted to generate a negative control staining. Sections were incubated with a biotinylated anti-BrdU antibody and subsequently with streptavidin-conjugated horseradish peroxidase, and staining reactions were developed using diaminobenzidine tetrahydrochloride according to the manufacturer's instructions. Nuclear counterstaining was performed with methyl green provided in the kit. Stained tissue sections were converted to digital images using a NanoZoomer Slide Scanner (Hamamatsu Photonics, Hamamatsu City, Shizuoka, Japan).

### Viruses

HIV-1_BaL_ was obtained through the NIH AIDS Reagent Program (Division of AIDS, NIAID, NIH) from Drs. Suzanne Gartner, Mikulas Popovic, and Robert Gallo [74,75]. A cell-free virus stock was produced in PBMCs. Culture supernatant was harvested at day 10 post-infection, passed through a 0.45μm filter, aliquoted and stored at -80°C.

Plasmids encoding the molecular clones pCH077.t/2627, pRHPA.c/2635, and pTHRO.c/2626 were obtained through the NIH AIDS Reagent Program, Division of AIDS, NIAID, NIH: Panel of full-length transmitted/founder (T/F) HIV-1 Infectious Molecular Clones (Cat #11919) from Dr. John Kappes and Dr. Christina Ochsenbauer [30,31,76–78]. The plasmids were amplified in One Shot Stbl3 Chemically Competent *E*. *coli* (ThermoFisher Scientific) and purified using the EndoFree Plasmid Maxi Kit (Qiagen) according to the manufacturer's instructions. Sequence homology between original and amplified plasmids was confirmed by digestion with restriction enzymes and gel electrophoresis. 293T cells were a kind gift of Mr. Vlad Radoi. Transfection of 293T cells with plasmids was carried out using FuGENE 6 Transfection Reagent (Promega, Madison WI, USA) according to the manufacturer's instructions. Culture supernatant was harvested at 48 and 72 h post-transfection, filtered, aliquoted and stored at -80°C. A cell-free viral stock was also produced by infecting PBMCs with the virus produced in 293T cells. Culture supernatant was harvested at day 8–10 post-infection, filtered, aliquoted and stored at -80°C.

### HIV-1 infection of ectocervical tissue explants and HIV-1 quantification

Donor-matched ectocervical explants mounted in agarose (3 explants per well) were incubated with CM or SP25% for 4 h at 37°C, CO_2_ 5%, and humidity 95% (S1B Fig). The supernatant was removed, and the explants washed with CM and incubated for 18 h at 37°C with a final volume of 400μl of HIV-1 stock. For T/F HIV-1, viral stocks produced in 293T cells and PBMCs were mixed 1:1 immediately before explant infection. The p24_gag_ concentration (ng/ml) of virus inoculum was 48.8 ± 5.3 for HIV-1_BaL_, 153.8 ± 6.7 for pCH077.t/2627, 179.0 ± 6.6 for pRHPA.c/2635, and 127.1 ± 2.6 for pTHRO.c/2626 (mean ± s.e.m., n = 3). Alternatively, donor-matched explants mounted in agarose were infected with 400μl of a mixture of HIV-1_BaL_ and SP25% (final concentration) or CM for 12 h at 37°C. The p24_gag_ concentration (ng/ml) of virus inoculum was 104.9 ± 14.9 (mean ± s.e.m, n = 3). Depending on the amount of tissue available, 9–12 explants were used for each experimental condition to generate two technical replicates.

### a) HIV-1 p24_gag_ immunoassay

After infection, the supernatant was removed and the explants were transferred onto gelatin sponges (Aegis Lifesciences, Ahmedabad, India) in a 12-well plate containing CM supplemented with FBS 15% (4–6 explants per well). The explants were maintained at the liquid-air interface for 18 days, as previously described [79,80]. Culture supernatant was sampled every 3 days before replacing it with fresh medium, and stored at -80°C. Viral replication was quantified by measuring the amount of HIV-1 p24_gag_ in culture medium using a bead-based immunoassay [81]. Cumulative virus production was calculated by summing the p24_gag_ concentrations measured over time. At the end of culture, infected tissue explants were cryopreserved at -80°C for DNA extraction or *in situ* HIV RNA hybridization.

### b) HIV-1 DNA quantitative real-time PCR

DNA was extracted from infected tissue explants using the DNeasy Blood and Tissue Kit (Qiagen) according to the manufacturer’s instructions. At least five tissue explants for each experimental condition (e.g. CM, SP) were processed to generate a DNA preparation. RNA digestion with RNase A was performed to avoid co-purification of viral RNA. Eluted DNA was diluted 1:4 in water. HIV-1 *pol* copy numbers were quantified by real-time qPCR as described by Gibellini et al [82]. The gene hemoglobin subunit beta (*HBB*) was used to normalize HIV-1 DNA copy numbers to the amount of input DNA in order to compare samples. The sequences of HIV-1 *pol* and *HBB* primers are listed in S3 Table [83,84]. Serial dilutions of the pTHRO.c/2626 plasmid and human DNA were used to generate standard curves to quantify HIV-1 *pol* and *HBB* copy numbers respectively, as determined by regression analysis of amplicon abundance vs. Ct values. Amplification efficiency was within 90 and 110% for all included assays. Amplification was performed in a 20μl-reaction containing HOT FIREPol EvaGreen qPCR Supermix (Solis BioDyne), forward and reverse primers 0.25μM each, and 10μl of DNA, using a QuantStudio 5 real-time PCR system (Applied Biosystems). The thermal cycler profile was as follows: 95°C for 12 min, 40 cycles of 95°C for 15 sec, 60°C for 30 sec, 72°C for 30 sec, 75°C for 5 sec (HIV-1 *pol*); 95°C for 12 min, 40 cycles of 95°C for 15 sec, 58°C for 30 sec, 72°C for 30 sec (*HBB*). PCR products were analyzed by dissociation curve profile. Samples were run in duplicate.

### c) HIV-1 RNA *in situ* hybridization

Cryopreserved samples of ectocervical tissue explants infected with HIV-1 were mounted in OCT (Histolab Products) and cut into 8μm-thick sections. Sections were fixed overnight in chilled PFA 4% (Sigma-Aldrich) at 4°C and processed to detect HIV RNA using the RNAscope technology [85], according to the RNAscope 2.0 HD Detection Kit (RED) (Histolab Products). The following probes (all from Histolab Products) were used: Probe Hs-HIV (311921), Positive Control Probe Hs-UBC (310041), Positive Control Probe Hs-PPIB (313901), and Negative Control Probe DapB (310043). Nuclear counterstaining was performed with hematoxylin (Histolab Products). Stained tissue sections were converted to digital images using a Panoramic 250 Flash Slide Scanner (3DHistech, Budapest, Hungary).

### Statistical analysis

All experiments were conducted independently using donor-matched tissue or ECM. To minimize the effects of biological variability between tissue donors and SP pools, the results of explant treatment with SP and/or indomethacin were normalized to those of donor-matched untreated control tissue (CM), and the ratio was defined as n-fold. N-fold values were tested against 1 using the Wilcoxon signed rank test. Differences in n-fold change between two groups were evaluated using the Mann-Whitney test for groups with mixed paired and unpaired data, and the Wilcoxon matched-pairs signed rank test for groups containing only paired data. All tests were two-tailed. Differences in ECM cytokine n-fold change between multiple groups were evaluated using the Kruskal-Wallis test for groups with mixed paired and unpaired data, and the Friedman test for groups containing only paired data, with Dunn's multiple comparisons test. p<0.05 indicated statistical significance.

## Accession numbers

Accession numbers (Entrez Gene) for the genes and transcripts analyzed here are reported in S3 Table.

Proteins (Swiss-Prot): IL-1α (P01583.1), IL-6 (Q75MH2), TNF (P01375.1), CCL5 (P13501.3), CCL20 (P78556.1), CXCL1 (P09341.1), CXCL8 (P10145.1), TGF-β1 (P01137.2), IL-10 (P22301.1).

Viruses (Entrez Nucleotide): HIV-1_BaL_ (AY713409.1), pCH077.t/2627 (JN944941), pRHPA.c/2635 (JN944944), pTHRO.c/2626 (JN944946).

## Supporting information

S1 Fig Study experimental design. **A)** Donor-matched explants of ectocervical mucosa were mounted in agarose medium with the epithelial side upwards, and incubated with seminal plasma (SP) or culture medium (CM) for 2, 4 and 12 h. Tissue integrity and cell viability were assessed by *in situ* TUNEL assay in explants harvested after 12 h-incubation with SP50% or CM. To measure cytokine expression, the supernatant was removed, and explants were washed with CM and incubated with fresh CM for 12 h. Explant conditioned medium (ECM) and explants were harvested for protein and gene expression analysis respectively. Cell viability was also evaluated on cells isolated from explants incubated in the presence or absence of SP50% for 12 h followed by an additional 12 h-incubation with CM. **B)** Explants mounted in agarose were incubated with CM or SP25% for 4 h. Explants were washed with CM and incubated with a suspension of cell-free HIV-1_BaL_ or transmitted/founder (T/F) HIV-1 for 18 h. Alternatively, explants were incubated with a mixture of HIV-1_BaL_ and SP25% (final concentration) or CM for 12 h. In both experimental settings, infected explants were washed and transferred onto gelatin sponges soaked in medium, and maintained at the liquid-air interface for 18 days with a change of medium every 3 days.
(TIF)

S2 Fig IL-6 and CXCL8 concentration in ectocervical tissue explant conditioned medium (ECM) of excluded experiments. **A)** Cytokine concentration (ng/ml) was measured in explant conditioned medium (ECM) of ectocervical explants incubated with culture medium (CM) or seminal plasma (SP) 50%, in the presence or absence of indomethacin (indo) 10μM for 4 or 12 h, followed by an additional 12 h-incubation with medium only. Lines connect measurements obtained from donor-matched explants (n = 7). CM (white), CM+indomethacin (gray), SP50% (red), and SP50%+indomethacin (brown). **B)** N-fold change in ECM cytokine concentration of explants treated with SP and/or indomethacin, compared to donor-matched untreated explants (CM). Bars indicate median values. p<0.05 denotes a significant difference with CM (Wilcoxon signed rank test).
(TIF)

S3 Fig Indomethacin titration. **A)** Cytokine concentration (ng/ml) was measured in explant conditioned medium (ECM) of ectocervical explants incubated with culture medium (CM, white), seminal plasma (SP, red) 50% or lipopolysaccharides (LPS, gray) 2μg/ml, in the presence or absence of indomethacin (indo) 10μM or 30μM, for 12 h, followed by an additional 12 h-incubation with medium only. Bars indicate mean with s.e.m. (n = 3–4). **B)** N-fold change in ECM cytokine concentration of explants treated with SP, LPS and/or indomethacin, compared to donor-matched untreated explants (CM). Bars indicate median values. p<0.05 denotes a significant difference between two groups (Friedman test with Dunn's multiple comparisons test).
(TIF)

S4 Fig Seminal cytokine carry-over in ectocervical tissue explant conditioned medium (ECM). Donor-matched ectocervical tissue explants were processed fresh or inactivated with paraformaldehyde (PFA), and incubated with culture medium (CM vs. CM-PFA) or seminal plasma (SP vs. SP-PFA) 50% for 12 h, followed by an additional 12 h-incubation with medium only. **A)** Cytokine concentration (pg/ml) was measured in explant culture medium collected before wash, immediately after wash, and ECM, and in the SP pools used to treat explants. Bars indicate mean with s.e.m. (n = 5). **B)** N-fold change in ECM cytokine concentration of SP-treated explants (SP) compared to donor-matched untreated explants (CM) (red). The same n-fold change was calculated using ECM cytokine concentration values of SP-treated explants (SP) corrected for seminal cytokine carry-over (SP-PFA) (black) by subtracting SP-PFA-values from SP-values. Bars indicate median values. p<0.05 denotes a significant difference between medians (Wilcoxon matched-pairs signed rank test).
(TIF)

S5 Fig Gating strategy used for flow cytometric analysis of peripheral blood leukocytes in transmigration assays. Acquired events were identified as: 1) counting beads; 2) leukocytes (CD45+); 3) granulocytes; 4) neutrophils (CD16+CD66b+); 5) T cells (CD16-CD3+); 6) classical monocytes (CD16-CD14+).
(TIF)

S6 Fig Expression levels of the chemokine receptors CXCR1 and CXCR2 on transmigrated granulocytes. Mononuclear cells and granulocytes were isolated from blood, pooled (i.e. peripheral blood leukocytes (PBL)) and incubated in a transwell system for 2 h with explant conditioned medium (ECM) from donor-matched ectocervical explants incubated with culture medium (CM-ECM) or seminal plasma (SP-ECM). Transmigrated cells were immunophenotyped and enumerated by flow cytometry (see S5 Fig). **A)** Peaks represent PBL untreated cultured (ctrl, gray), cultured with SP-ECM (SP, red), cultured with CM-ECM (CM, white), and unstained control (black) from one representative experiment. **B)** CXCR1 and CXCR2 mean fluorescence intensity (MFI). Bars indicate median values. **C)** N-fold change in CXCR1 and CXCR2 MFI on PBL cultured with ECM compared to ctrl. Lines connect measurements obtained from donor-matched ECM. p<0.05 denotes a significant difference with ctrl (Wilcoxon signed rank test).
(TIF)

S7 Fig Cell viability and tissue architecture of cultured ectocervical explants. Sections were obtained from donor-matched ectocervical tissue explants: **A** and **D**) incubated seminal plasma (SP) 50% for 12 h; **B**) incubated with culture medium (CM) for 12 h; **C**) snap-frozen immediately after dissection. **A**, **B**, and **C**) Sections were processed to reveal chromatin fragmentation as a marker of apoptosis with a TUNEL assay. **D**) The staining negative control was generated by omitting the TdT enzyme. Data are from one representative of three independent experiments. Apoptotic cells are shown in brown, and are indicated by arrows in panel C. Sections were counterstained with methyl green. Scale bars = 100μm.
(TIF)

S8 Fig Effect of progesterone-based drug use on HIV-1_BaL_ infection. Infection of ectocervical explants with HIV-1_BaL_ was independently performed after an initial treatment with seminal plasma (post-SP, red), or in the presence of SP (SP-mix, blue) (see S1B Fig). Experiments were grouped based on progesterone-based drug use of tissue donors. N-fold change in cumulative p24_gag_ production in SP-treated explants compared to donor-matched untreated explants (CM). Bars indicate median values. p<0.05 denotes a significant difference between groups (Mann-Whitney test).
(TIF)

S9 Fig Imaging of HIV-1 replication in ectocervical tissue explants. Representative section of a tissue explant infected with HIV-1_BaL_ harvested on day 18 post-infection (top) and uninfected uncultured ectocervical tissue (bottom). *In situ* HIV RNA hybridization signal is shown in red. Scale bars = 200μm; 50μm (magnification).
(TIF)

S1 Table Cytokine concentration in ectocervical tissue explant conditioned medium (ECM) and seminal plasma pools. Cytokine concentrations (pg/ml) was measured in ECM of donor-matched ectocervical explants incubated with culture medium (CM), seminal plasma (SP) 25% or SP50%, in the presence or absence of indomethacin 10μM, for 2, 4 and 12 h. On the right, cytokine concentrations (pg/ml) in SP pools used to treat explants. Concentration values are reported as median and IQR. Values of n-fold change in ECM cytokine concentrations of explants treated with SP and/or indomethacin compared to donor-matched untreated explants (CM) are reported as median and IQR. p<0.05 (in bold) indicates a statistically significant difference with CM (Wilcoxon signed rank test).
N/A not applicable.
^a^ n = 7 for 2h incubation; n = 11 for 4h and 12h incubations.
^b^ more than 50% of values were below the lower limit of quantification.
(PDF)

S2 Table Gene expression in ectocervical tissue explants. N-fold change in gene expression measured in ectocervical explants incubated with seminal plasma (SP) 50% and/or indomethacin 10μM for 4 and 12 h, compared to donor-matched untreated explants (CM). Values are reported as median and IQR (n = 7). P<0.05 (in bold) indicates a statistically significant difference with CM (Wilcoxon signed rank test).
(PDF)

S3 Table Primers used for quantitative real-time PCR. N/A not applicable.
(PDF)
