# Correction: Seminal plasma induces inflammation and enhances HIV-1 replication in human cervical tissue explants

**DOI:** 10.1371/journal.ppat.1006492

**Published:** 2017-07-11

**Authors:** Andrea Introini, Stéphanie Boström, Frideborg Bradley, Anna Gibbs, Axel Glaessgen, Annelie Tjernlund, Kristina Broliden

In panel A of Fig 3, the gray and white bars in the chart are filled incorrectly. The white bars should be gray and the gray bars should be white. Please see the corrected Fig 3 here.

**Fig 3:**
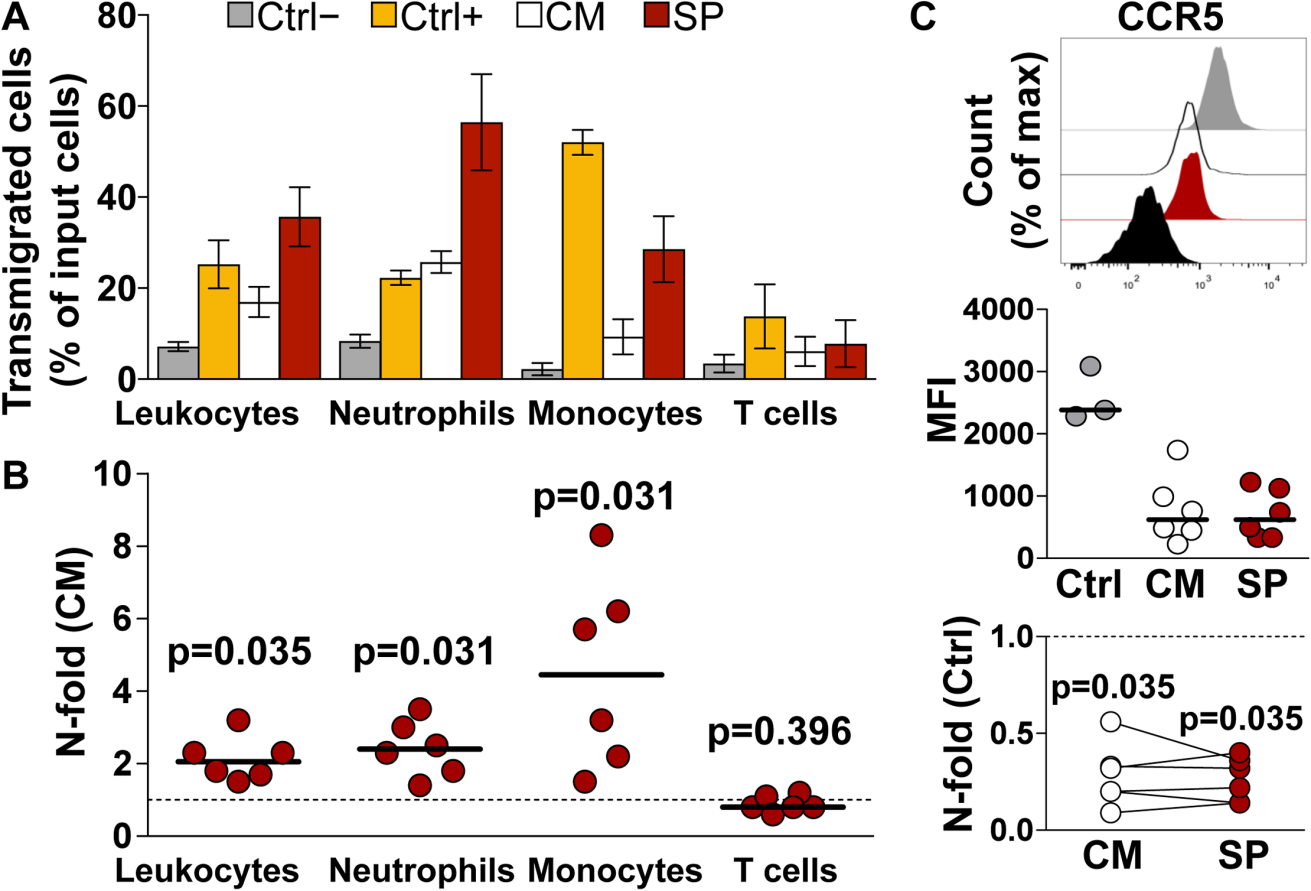
Transwell migration of peripheral blood leukocytes (PBL). Mononuclear cells and granulocytes were isolated from blood, pooled (i.e. PBL) and incubated in a transwell system for 2 h with explant conditioned medium (ECM) from donor-matched ectocervical explants incubated with culture medium (CM-ECM) or seminal plasma (SP-ECM). Transmigrated cells were immunophenotyped and enumerated by flow cytometry (see S5 Fig). **A)** Fraction of transmigrated cells out of total number of PBL loaded into a transwell insert for each analyzed cell population (input). PBL were incubated with CM-ECM (white), SP-ECM (red), and with medium only (grey) and medium supplemented with FBS 10% (yellow) as negative and positive controls respectively. Bars represent mean with s.e.m (n = 6). **B)** N-fold change in the fraction of transmigrated PBL incubated with SP-ECM compared to that of donor-matched CM-ECM. Bars indicate median values. p<0.05 denotes a significant difference with CM (Wilcoxon signed rank test). **C)** Expression levels of the chemokine receptor CCR5 on transmigrated monocytes. Top, peaks represent PBL untreated cultured (ctrl, gray), cultured with CM-ECM (CM, white), cultured with SP-ECM (SP, red), and unstained control (black) from one representative experiment. Middle, CCR5 mean fluorescence intensity (MFI). Bars indicate median values. Bottom, n-fold change in CCR5 MFI on PBL cultured with ECM compared to untreated cultured PBL (ctrl). Lines connect measurements obtained from donor-matched ECM. p<0.05 denotes a significant difference with ctrl (Wilcoxon signed rank test).
