# Supplementary figures and images for: Seminal plasma induces inflammation and enhances HIV-1 replication in human cervical tissue explants
Source: PLoS Pathog. 2017 May 19;13(5):e1006402. doi: 10.1371/journal.ppat.1006402 (PMC5453613; doi:10.1371/journal.ppat.1006402)

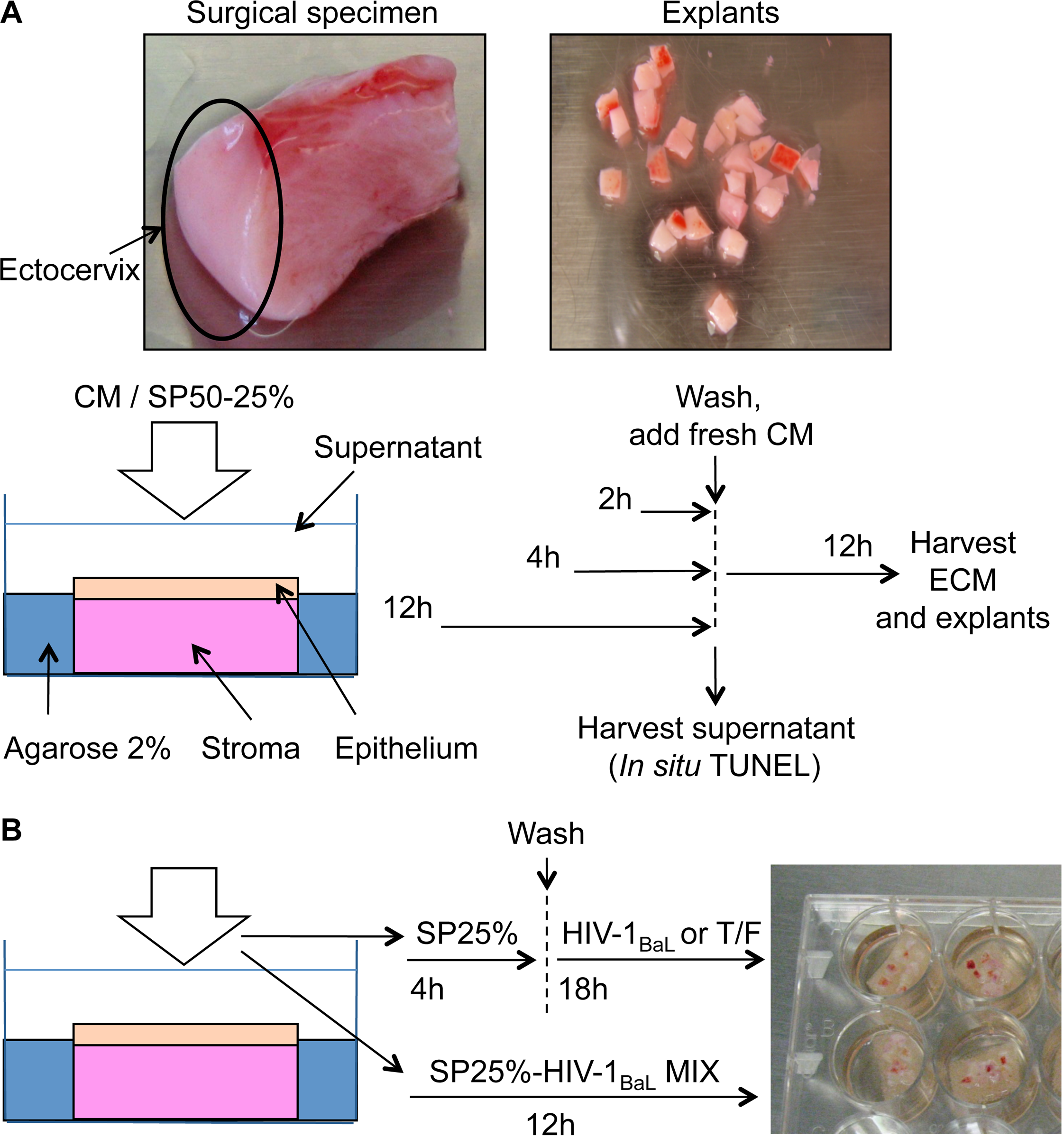

Supplement: S1 Fig — A) Donor-matched explants of ectocervical mucosa were mounted in agarose medium with the epithelial side upwards, and incubated with seminal plasma (SP) or culture medium (CM) for 2, 4 and 12 h. Tissue integrity and cell viability were assessed by in situ TUNEL assay in explants harvested after 12 h-incubation with SP50% or CM. To measure cytokine expression, the supernatant was removed, and explants were washed with CM and incubated with fresh CM for 12 h. Explant conditioned medium (ECM) and explants were harvested for protein and gene expression analysis respectively. Cell viability was also evaluated on cells isolated from explants incubated in the presence or absence of SP50% for 12 h followed by an additional 12 h-incubation with CM. B) Explants mounted in agarose were incubated with CM or SP25% for 4 h. Explants were washed with CM and incubated with a suspension of cell-free HIV-1BaL or transmitted/founder (T/F) HIV-1 for 18 h. Alternatively, explants were incubated with a mixture of HIV-1BaL and SP25% (final concentration) or CM for 12 h. In both experimental settings, infected explants were washed and transferred onto gelatin sponges soaked in medium, and maintained at the liquid-air interface for 18 days with a change of medium every 3 days. (TIF) [file ppat.1006402.s001.tif]

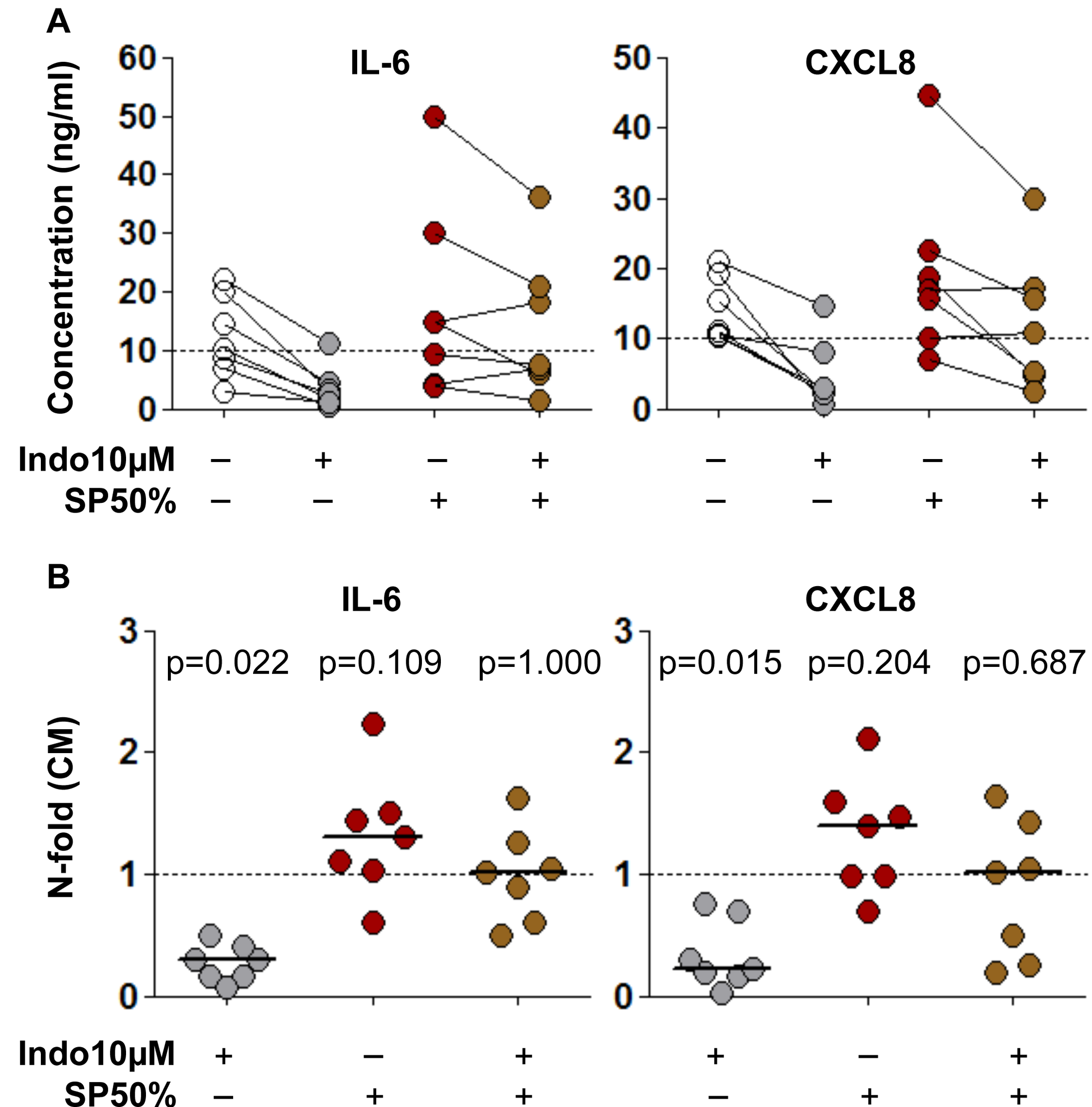

Supplement: S2 Fig — A) Cytokine concentration (ng/ml) was measured in explant conditioned medium (ECM) of ectocervical explants incubated with culture medium (CM) or seminal plasma (SP) 50%, in the presence or absence of indomethacin (indo) 10μM for 4 or 12 h, followed by an additional 12 h-incubation with medium only. Lines connect measurements obtained from donor-matched explants (n = 7). CM (white), CM+indomethacin (gray), SP50% (red), and SP50%+indomethacin (brown). B) N-fold change in ECM cytokine concentration of explants treated with SP and/or indomethacin, compared to donor-matched untreated explants (CM). Bars indicate median values. p<0.05 denotes a significant difference with CM (Wilcoxon signed rank test). (TIF) [file ppat.1006402.s002.tif]

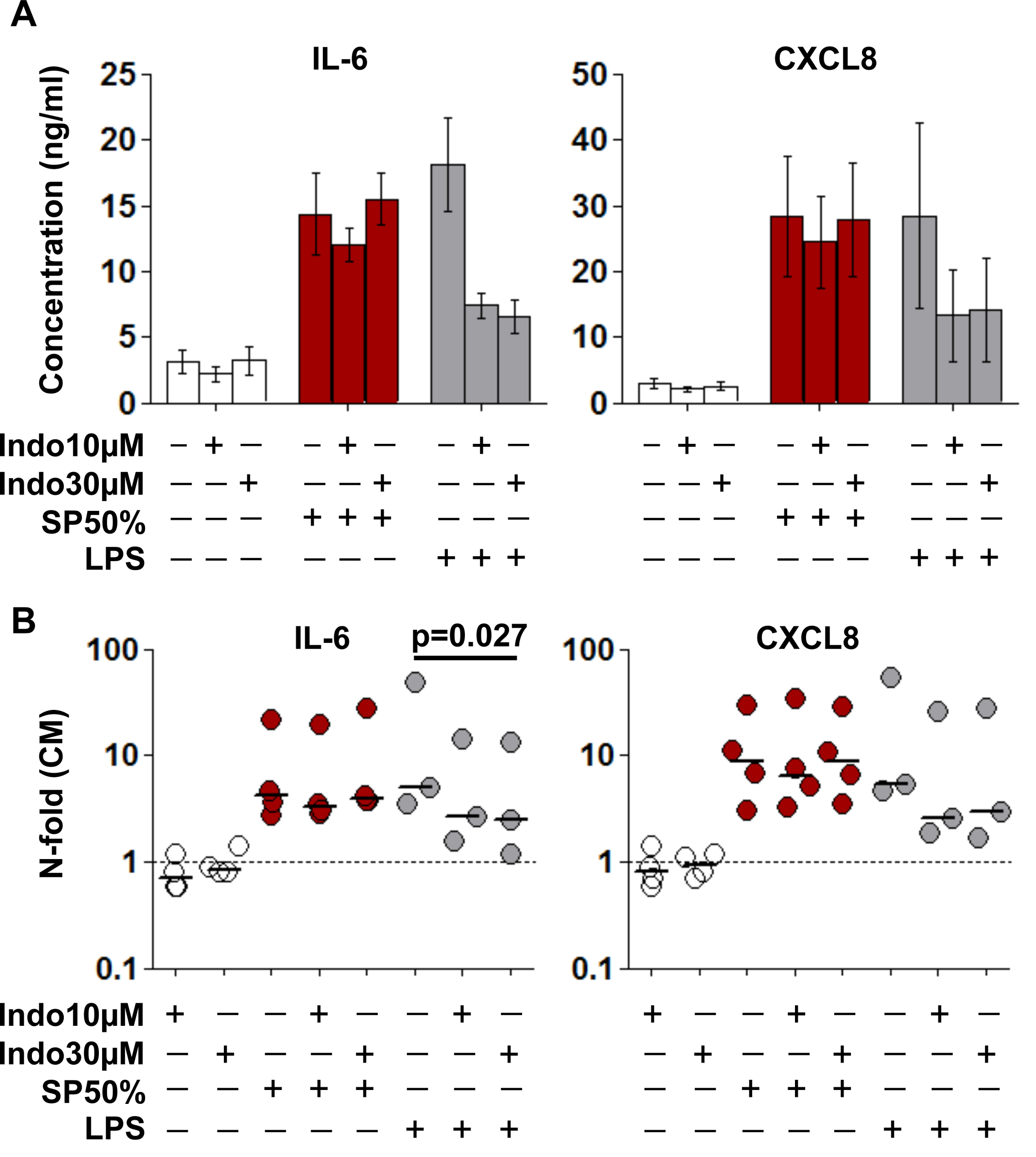

Supplement: S3 Fig — A) Cytokine concentration (ng/ml) was measured in explant conditioned medium (ECM) of ectocervical explants incubated with culture medium (CM, white), seminal plasma (SP, red) 50% or lipopolysaccharides (LPS, gray) 2μg/ml, in the presence or absence of indomethacin (indo) 10μM or 30μM, for 12 h, followed by an additional 12 h-incubation with medium only. Bars indicate mean with s.e.m. (n = 3–4). B) N-fold change in ECM cytokine concentration of explants treated with SP, LPS and/or indomethacin, compared to donor-matched untreated explants (CM). Bars indicate median values. p<0.05 denotes a significant difference between two groups (Friedman test with Dunn's multiple comparisons test). (TIF) [file ppat.1006402.s003.tif]

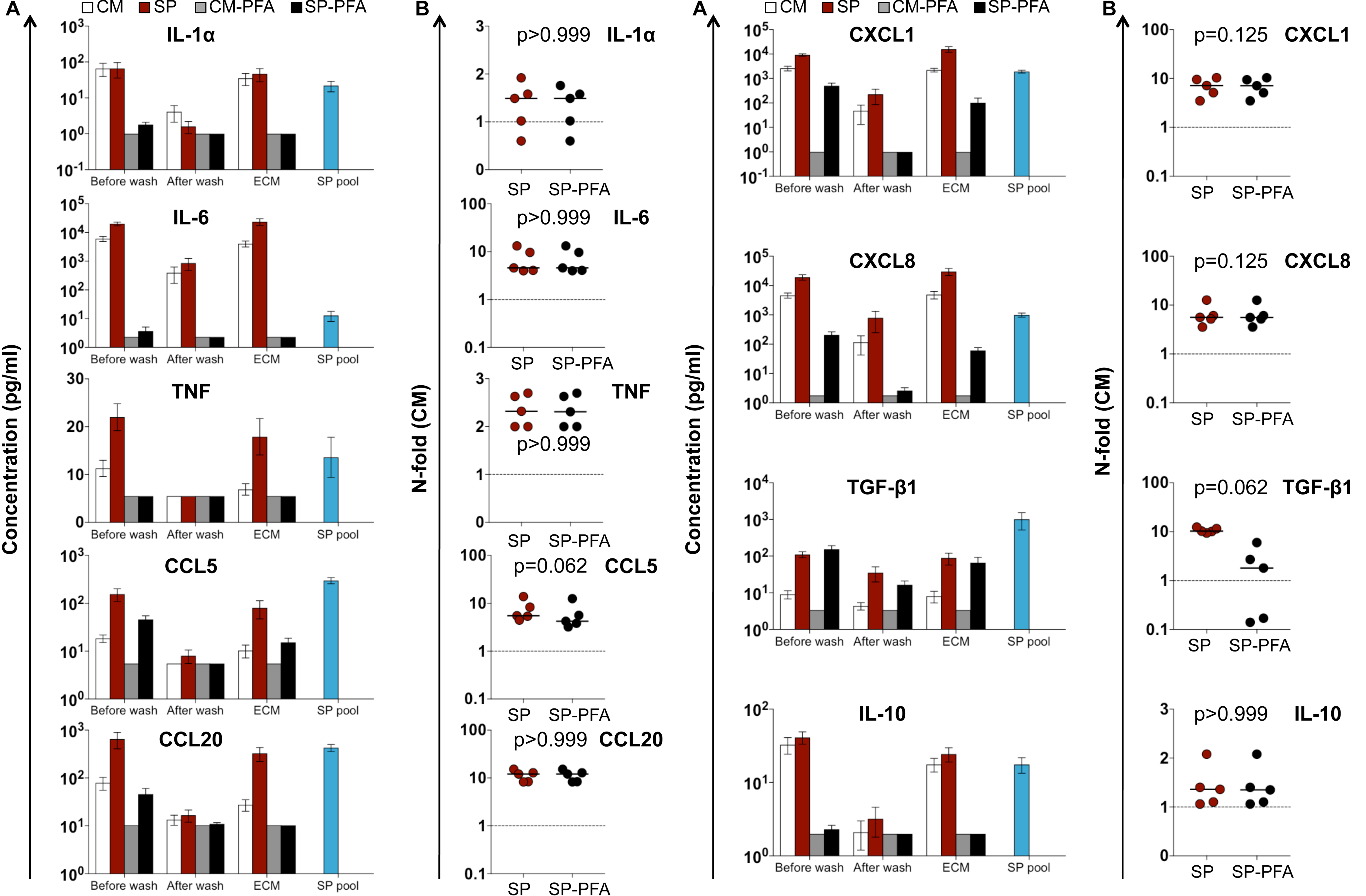

Supplement: S4 Fig — Donor-matched ectocervical tissue explants were processed fresh or inactivated with paraformaldehyde (PFA), and incubated with culture medium (CM vs. CM-PFA) or seminal plasma (SP vs. SP-PFA) 50% for 12 h, followed by an additional 12 h-incubation with medium only. A) Cytokine concentration (pg/ml) was measured in explant culture medium collected before wash, immediately after wash, and ECM, and in the SP pools used to treat explants. Bars indicate mean with s.e.m. (n = 5). B) N-fold change in ECM cytokine concentration of SP-treated explants (SP) compared to donor-matched untreated explants (CM) (red). The same n-fold change was calculated using ECM cytokine concentration values of SP-treated explants (SP) corrected for seminal cytokine carry-over (SP-PFA) (black) by subtracting SP-PFA-values from SP-values. Bars indicate median values. p<0.05 denotes a significant difference between medians (Wilcoxon matched-pairs signed rank test). (TIF) [file ppat.1006402.s004.tif]

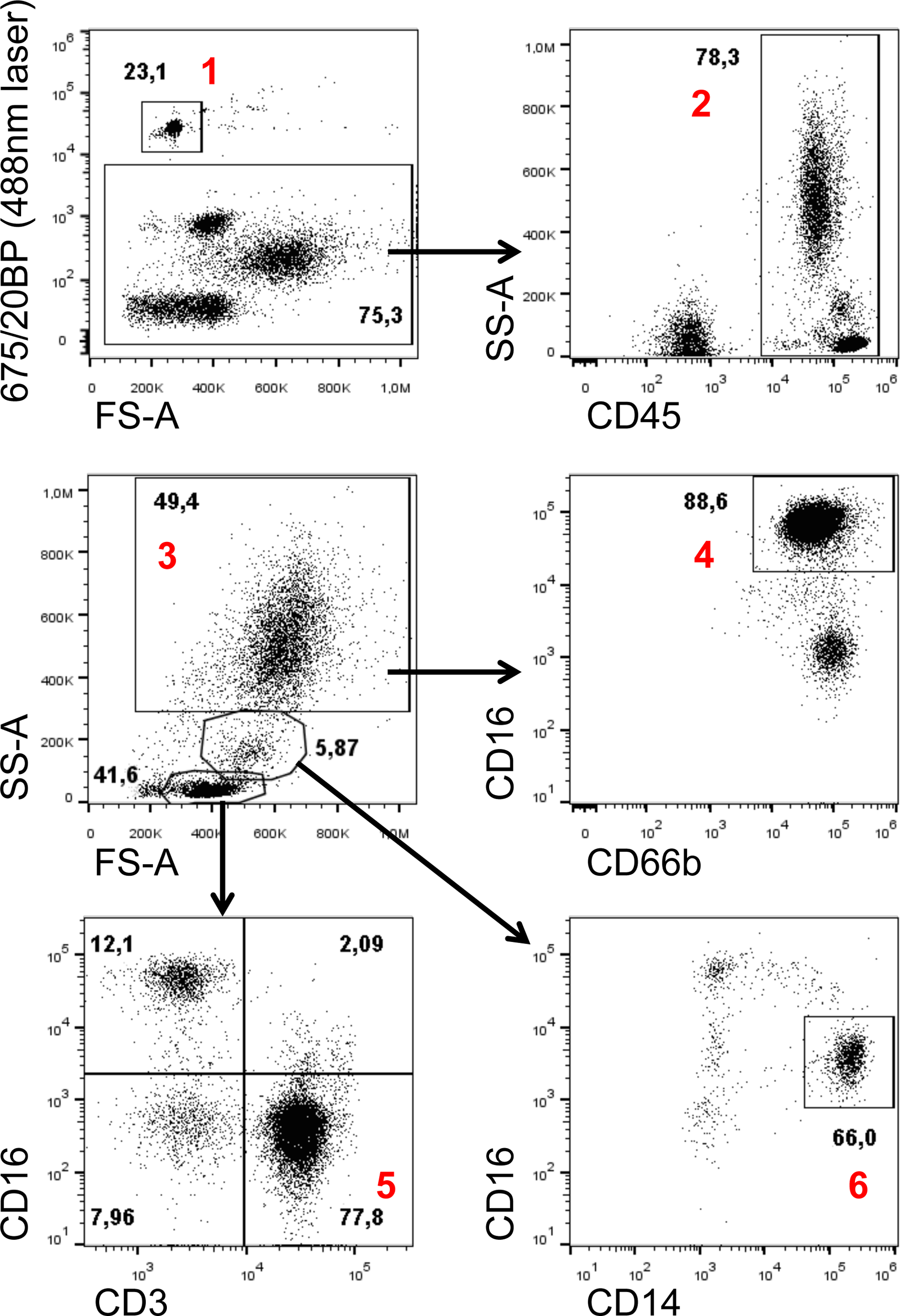

Supplement: S5 Fig — Acquired events were identified as: 1) counting beads; 2) leukocytes (CD45+); 3) granulocytes; 4) neutrophils (CD16+CD66b+); 5) T cells (CD16-CD3+); 6) classical monocytes (CD16-CD14+). (TIF) [file ppat.1006402.s005.tif]

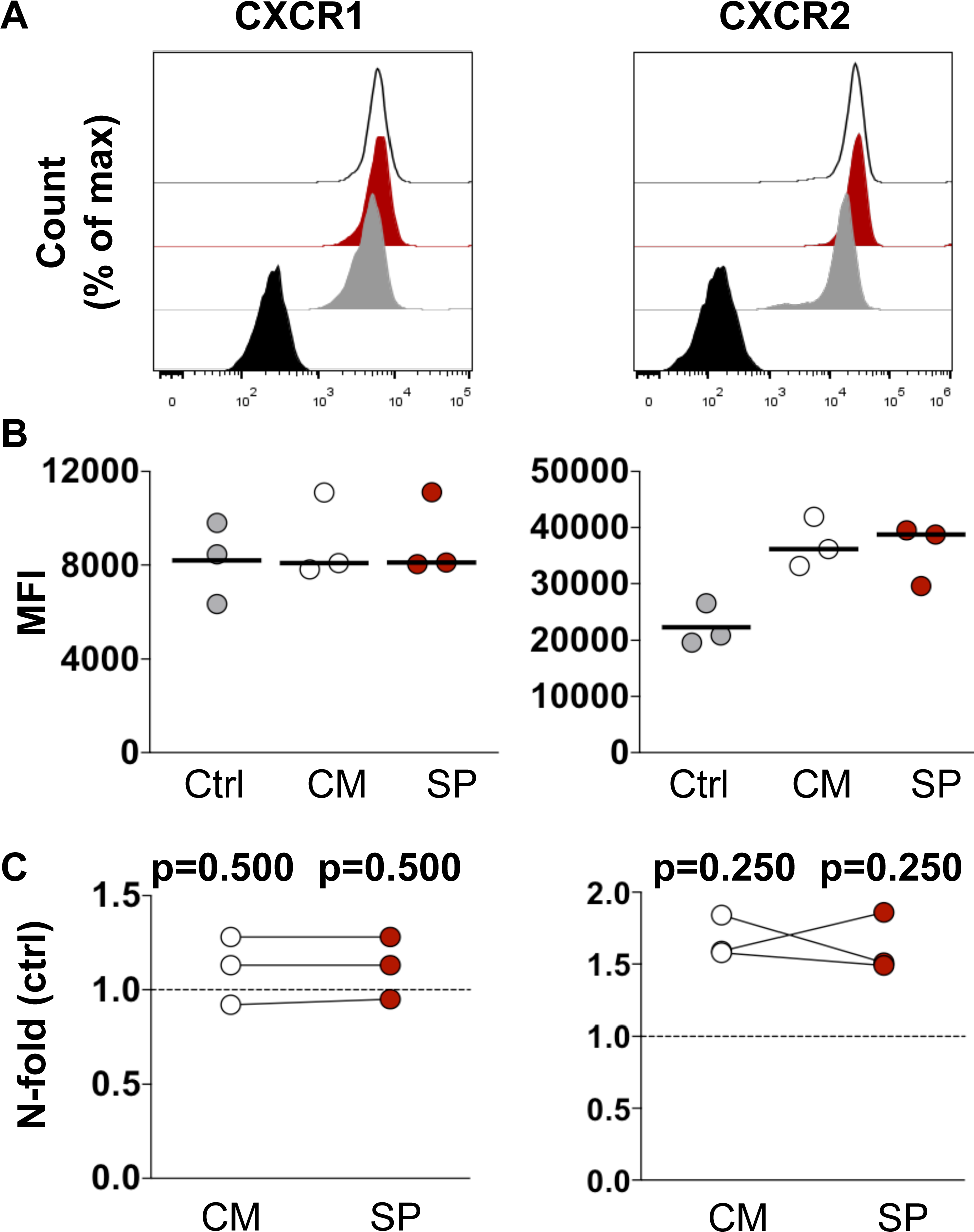

Supplement: S6 Fig — Mononuclear cells and granulocytes were isolated from blood, pooled (i.e. peripheral blood leukocytes (PBL)) and incubated in a transwell system for 2 h with explant conditioned medium (ECM) from donor-matched ectocervical explants incubated with culture medium (CM-ECM) or seminal plasma (SP-ECM). Transmigrated cells were immunophenotyped and enumerated by flow cytometry (see S5 Fig). A) Peaks represent PBL untreated cultured (ctrl, gray), cultured with SP-ECM (SP, red), cultured with CM-ECM (CM, white), and unstained control (black) from one representative experiment. B) CXCR1 and CXCR2 mean fluorescence intensity (MFI). Bars indicate median values. C) N-fold change in CXCR1 and CXCR2 MFI on PBL cultured with ECM compared to ctrl. Lines connect measurements obtained from donor-matched ECM. p<0.05 denotes a significant difference with ctrl (Wilcoxon signed rank test). (TIF) [file ppat.1006402.s006.tif]

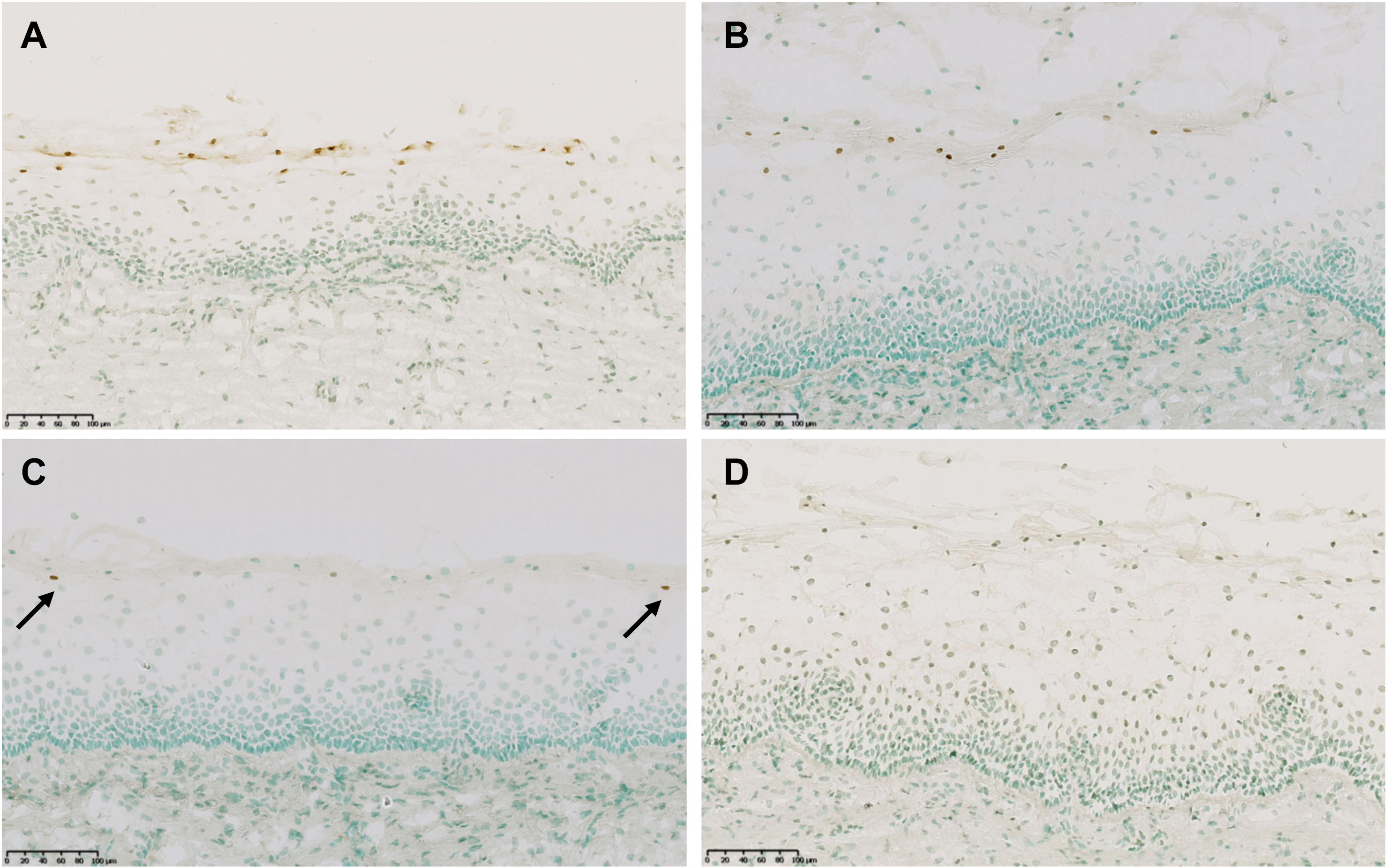

Supplement: S7 Fig — Sections were obtained from donor-matched ectocervical tissue explants: A and D) incubated seminal plasma (SP) 50% for 12 h; B) incubated with culture medium (CM) for 12 h; C) snap-frozen immediately after dissection. A, B, and C) Sections were processed to reveal chromatin fragmentation as a marker of apoptosis with a TUNEL assay. D) The staining negative control was generated by omitting the TdT enzyme. Data are from one representative of three independent experiments. Apoptotic cells are shown in brown, and are indicated by arrows in panel C. Sections were counterstained with methyl green. Scale bars = 100μm. (TIF) [file ppat.1006402.s007.tif]

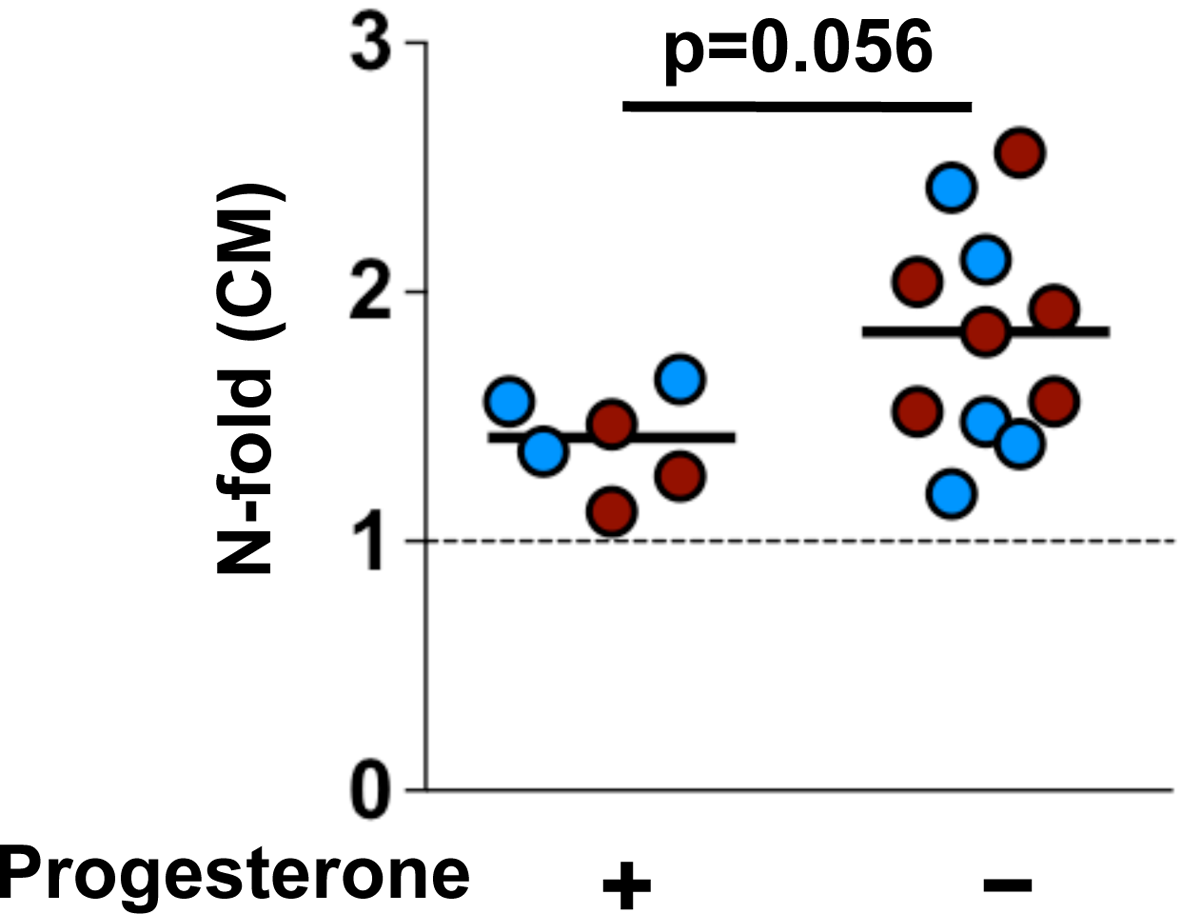

Supplement: S8 Fig — Infection of ectocervical explants with HIV-1BaL was independently performed after an initial treatment with seminal plasma (post-SP, red), or in the presence of SP (SP-mix, blue) (see S1B Fig). Experiments were grouped based on progesterone-based drug use of tissue donors. N-fold change in cumulative p24gag production in SP-treated explants compared to donor-matched untreated explants (CM). Bars indicate median values. p<0.05 denotes a significant difference between groups (Mann-Whitney test). (TIF) [file ppat.1006402.s008.tif]

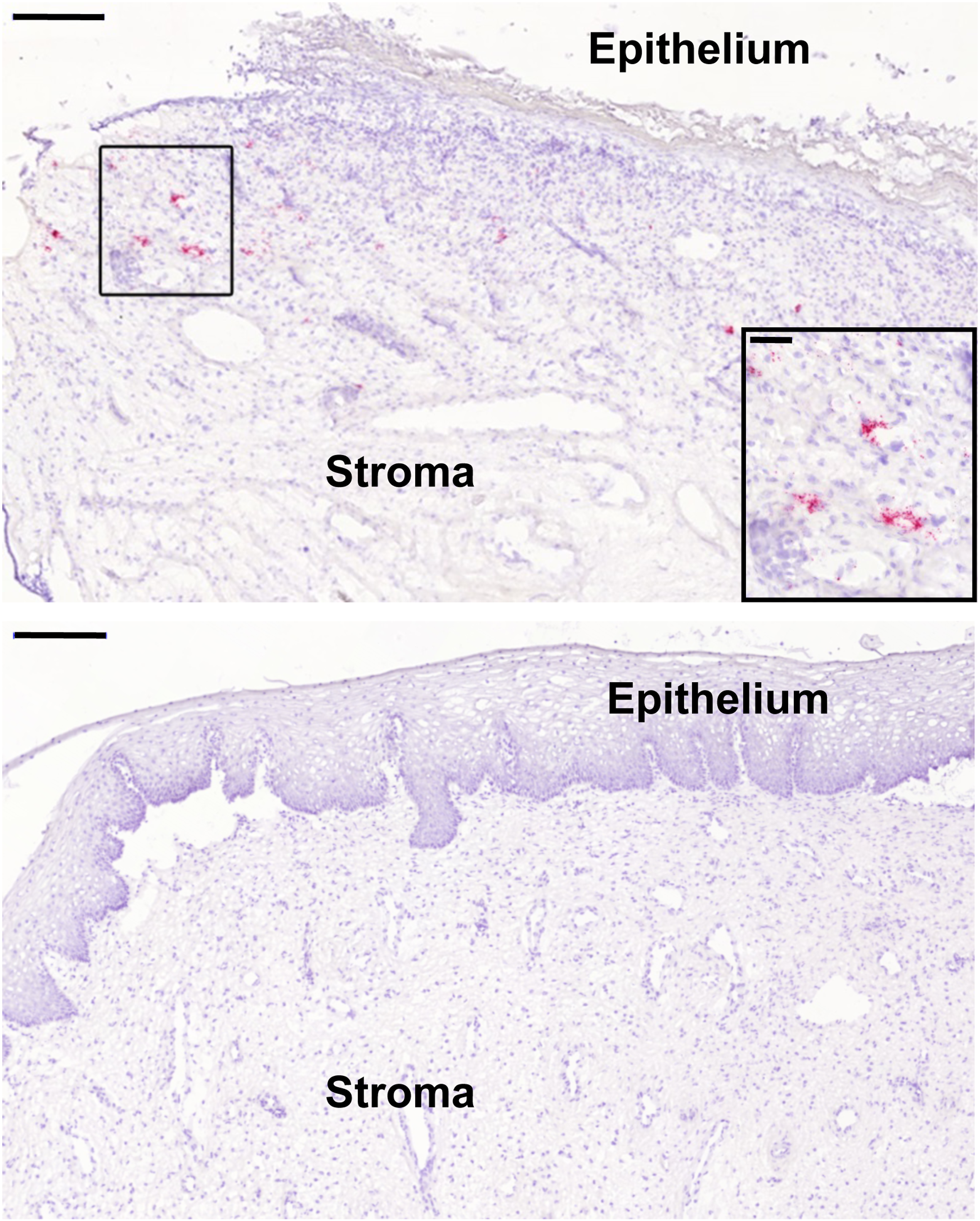

Supplement: S9 Fig — Representative section of a tissue explant infected with HIV-1BaL harvested on day 18 post-infection (top) and uninfected uncultured ectocervical tissue (bottom). In situ HIV RNA hybridization signal is shown in red. Scale bars = 200μm; 50μm (magnification). (TIF) [file ppat.1006402.s009.tif]
