# Supplementary material for: Seminal plasma induces inflammation and enhances HIV-1 replication in human cervical tissue explants
Source: PLoS Pathog. 2017 May 19;13(5):e1006402. doi: 10.1371/journal.ppat.1006402 (PMC5453613; doi:10.1371/journal.ppat.1006402)
Supplement: S1 Table — Cytokine concentrations (pg/ml) was measured in ECM of donor-matched ectocervical explants incubated with culture medium (CM), seminal plasma (SP) 25% or SP50%, in the presence or absence of indomethacin 10μM, for 2, 4 and 12 h. On the right, cytokine concentrations (pg/ml) in SP pools used to treat explants. Concentration values are reported as median and IQR. Values of n-fold change in ECM cytokine concentrations of explants treated with SP and/or indomethacin compared to donor-matched untreated explants (CM) are reported as median and IQR. p<0.05 (in bold) indicates a statistically significant difference with CM (Wilcoxon signed rank test). N/A not applicable. a n = 7 for 2h incubation; n = 11 for 4h and 12h incubations. b more than 50% of values were below the lower limit of quantification. (PDF) [file ppat.1006402.s010.pdf]

| | | CM (n = 7-11) [a] | CM + indomethacin (n = 7) | | | SP25% (n = 7) | | | SP50% (n = 7-11) [a] | | | SP50% + indomethacin (n = 7) | | | SP pool (n = 5) |
|---|---|---|---|---|---|---|---|---|---|---|---|---|---|---|---|
| Cytokine | Time (h) | Concentration (pg/ml) | Concentration (pg/ml) | N-fold (CM) | p-value | Concentration (pg/ml) | N-fold (CM) | p-value | Concentration (pg/ml) | N-fold (CM) | p-value | Concentration (pg/ml) | N-fold (CM) | p-value | Concentration (pg/ml) |
| IL-1α | 2 | 8.9 [4.0 - 24.2] | N/A | N/A | N/A | 11.6 [3.9 - 53.5] | 1.5 [1.0 - 2.0] | **0.02** | 16.3 [8.4 - 28.9] | 1.0 [0.9 - 3.2] | 0.62 | N/A | N/A | N/A | 11.4 [8.4 - 23.5] |
| | 4 | 15.6 [4.7 - 45.1] | 12.7 [6.9 - 27.3] | 0.9 [0.6 - 2.2] | >0.99 | 18.1 [3.2 - 124.3] | 1.0 [0.7 - 1.4] | 0.62 | 19.2 [5.1 - 37.5] | 1.1 [0.5 - 2.4] | 0.57 | 14.2 [6.9 - 45] | 1.3 [0.9 - 2.4] | 0.31 | |
| | 12 | 13.6 [6.6 - 24.8] | 10.4 [6.1 - 15.7] | 1.0 [0.8 - 1.4] | 0.87 | 30.8 [5.9 - 61.2] | 1.4 [1.2 - 3.3] | **0.03** | 13.9 [7.7 - 59.4] | 1.5 [0.7 - 3.0] | 0.07 | 9.3 [4.2 - 17.0] | 0.9 [0.3 - 1.7] | 0.78 | |
| IL-6 | 2 | 1513.5 [719.9 - 2807.2] | N/A | N/A | N/A | 7942.9 [3946.4- 9811.2] | 5.5 [2.1 - 6.7] | **0.01** | 10813.5 [5017.7- 14732.1] | 5.3 [3.8 - 8.4] | **0.01** | N/A | N/A | N/A | 78.6 [37.2 - 140.7] |
| | 4 | 1521.2 [921.9 - 5426.7] | 1227.5 [680.6 - 2052.5] | 0.7 [0.3 - 1.7] | 0.93 | 11117.2 [5923.8 - 13411.3] | 5.1 [3.4 - 6.1] | **0.01** | 12132.5 [7022.4 -15630.1] | 6.0 [3.2 - 8.9] | **0.001** | 13912.4 [7506.7 - 17237.3] | 6.6 [3.6 - 18.7] | **0.01** | |
| | 12 | 1309.6 [816.9 - 3387.0] | 1604.0 [1276.3 - 3800.1] | 1.7 [1.1 - 1.9] | **0.03** | 11245.9 [7517.0 - 18600.2] | 6.9 [3.5 - 15.6] | **0.01** | 16130.7 [10421.2 - 23501.7] | 12.3 [9.0 -15.7] | **0.001** | 13737.3 [8283.2 -15869.0] | 10.6 [6.8 - 13.9] | **0.01** | |
| TNF | 2 | 5.5 [5.5 - 5.6] [b] | N/A | N/A | N/A | 9.9 [6.1 - 13.0] | 1.8 [1.1 - 2.3] | **0.03** | 11.6 [9.1 - 19.7] | 2.1 [1.7 - 3.6] | **0.01** | N/A | N/A | N/A | 27.8 [25.3 - 71.2] |
| | 4 | 5.5 [5.5 - 6.6] [b] | 5.5 [5.5 - 7.9] [b] | 1.0 [0.8 - 1.0] | 0.75 | 12.5 [5.5 - 15.3] | 1.5 [1.0 - 2.5] | 0.12 | 18.8 [14.6 - 23.6] | 2.6 [2.0 - 4.0] | **0.02** | 12.0 [11.1 - 16.6] | 1.9 [1.1 - 3.0] | **0.03** | |
| | 12 | 5.5 [5.5 - 7.9] [b] | 5.5 [5.5 - 6.1] [b] | 1.0 [1.0 - 1.1] | >0.99 | 12.8 [5.8 - 18.8] | 2.0 [1.1 - 2.8] | **0.03** | 19.7 [11.1 - 21.9] | 2.9 [1.8 - 3.7] | **0.001** | 13.4 [11.0 - 24.6] | 2.4 [1.3 - 4.5] | **0.01** | |
| CCL5 (RANTES) | 2 | 6.5 [5.5 - 26.8] | N/A | N/A | N/A | 28.8 [16.4 - 123.3] | 3.0 [1.6 - 5.2] | **0.01** | 98.1 [11.5 - 228.2] | 4.6 [2.0 - 17.8] | **0.01** | N/A | N/A | N/A | 603.6 [517.1- 1492.0] |
| | 4 | 5.5 [5.5 - 7.8] [b] | 5.5 [5.5 - 10.0] [b] | 1.0 [1.0 - 1.5] | 0.50 | 36.5 [15.2 - 163.0] | 5.6 [1.8 - 13.8] | **0.03** | 86.1 [33.0 - 245.8] | 11.6 [5.5 - 25.7] | **0.001** | 79.9 [46.9 - 153.5] | 9.7 [5.2 - 19.6] | **0.01** | |
| | 12 | 5.5 [5.5 - 9.1] [b] | 6.3 [5.5 - 8.7] | 1.0 [1.0 - 1.2] | 0.62 | 40.7 [17.3 - 140.6] | 4.5 [3.1 - 5.9] | **0.01** | 73.6 [44.2 - 198.4] | 8.0 [4.0 - 16.0] | **0.001** | 83.4 [38.4 - 166.2] | 8.0 [5.2 - 24.9] | **0.01** | |
| CCL20 (MIP-3α) | 2 | 16.9 [10.3 - 19.6] | N/A | N/A | N/A | 92.9 [66.3 - 135.3] | 6.4 [2.6 - 13.1] | **0.01** | 107.8 [99.8 - 145.2] | 9.7 [5.9 - 10.7] | **0.01** | N/A | N/A | N/A | 555.4 [473.1- 1170.3] |
| | 4 | 10.3 [10.3-36.2] [b] | 10.3 [10.3-10.3] [b] | 1.0 [0.6 - 1.0] | 0.50 | 145.2 [57.3 - 180.4] | 4.3 [2.8 - 5.3] | **0.01** | 134.6 [39.3 - 212.7] | 3.8 [3.0 - 10.3] | **0.001** | 118.0 [91.0 - 152.2] | 8.8 [2.7 - 14.8] | **0.01** | |
| | 12 | 10.3 [10.3-21.4] [b] | 10.3 [10.3-10.3] [b] | 1.0 [0.7 - 1.0] | 0.34 | 90.9 [68.6 - 152.3] | 6.7 [1.9 - 6.9] | **0.01** | 123.3 [90.8 - 233.3] | 11.4 [6.8 - 16.1] | **0.002** | 91.1 [69.8 - 127.9] | 8.8 [6.5 - 12.4] | **0.01** | |
| CXCL1 (GROα) | 2 | 333.0 [268.1 - 363.1] | N/A | N/A | N/A | 2227.7 [998.1 - 4053.6] | 5.8 [3.1 - 11.2] | **0.01** | 4572.0 [3163.2 - 7077.1] | 11.0 [10.0 - 26.4] | **0.01** | N/A | N/A | N/A | 3595.4 [2451.3 - 9487.2] |
| | 4 | 475.9 [275.9 - 998.3] | 403.0 [283.9 - 958.2] | 0.6 [0.5 - 1.0] | 0.20 | 3225.0 [1821.5 - 3972.9] | 3.3 [2.1 - 12.6] | **0.01** | 3288.1 [3140.1 - 6032.3] | 7.1 [3.2 - 14.0] | **0.001** | 3776.1 [2680.9 - 5480.7] | 7.9 [2.7 - 19.6] | **0.01** | |
| | 12 | 631.8 [333.8 - 833.6] | 716.3 [562.8 - 1045.3] | 1.7 [1.4 - 2.7] | **0.03** | 4375.1 [1260.9 - 7720.7] | 5.8 [3.2 - 12.3] | **0.03** | 4993.1 [4319.5 - 9953.6] | 12.5 [6.3 - 13.8] | **0.003** | 4600.5 [3766.4 - 5981.5] | 10.1 [6.3 - 17.9] | **0.01** | |
| CXCL8 (IL-8) | 2 | 835.9 [594.3 - 1479.6] | N/A | N/A | N/A | 3469,7 [1963,8 - 10200,3] | 5.8 [1.5 - 8.5] | **0.01** | 4409.1 [2771.5 - 10370.2] | 5.1 [4.0 - 11.9] | **0.01** | N/A | N/A | N/A | 3159.1 [2035.2 - 5206.7] |
| | 4 | 1339.6 [738.1 - 2560.3] | 1019.8 [830.1 - 2743.4] | 0.7 [0.5 - 1.2] | 0.30 | 4804.0 [3242.8 - 9812.2] | 4.3 [2.3 - 7.1] | **0.02** | 10200.5 [5893.2 - 12635.2] | 7.6 [3.3 - 13.2] | **0.003** | 13840.7 [7449.4 - 18475.8] | 5.6 [3.6 - 16.3] | **0.01** | |
| | 12 | 1412.6 [977.6 - 2357.0] | 2262.8 [1496.2 - 3779.6] | 1.6 [1.1 - 2.0] | **0.02** | 13488.9 [5591.1 - 20000.0] | 5.8 [3.3 - 14.2] | **0.01** | 18430.0 [11829.8 - 22397.7] | 10.6 [7.9 - 18.8] | **0.001** | 16519.7 [11240.4 - 21256.6] | 11.7 [5.5 -21.4] | **0.01** | |
| TGF-β1 | 2 | 3.4 [3.4 - 3.4] [b] | N/A | N/A | N/A | 85.2 [49.6 - 255.4] | 25.1 [14. -75.1] | **0.01** | 155.8 [87.4 - 280.9] | 45.8 [25.7 - 82.6] | **0.01** | N/A | N/A | N/A | 602.4 [348.8 - 1022.6] |
| | 4 | 3.4 [3.4 - 3.4] [b] | 3.4 [3.4 - 6.3] [b] | 1.0 [0.5 - 1.0] | 0.50 | 97.2 [79.5 - 282.7] | 28.6 [15.0-89.8] | **0.01** | 179.3 [95.7 - 290.6] | 28.2 [14.1 - 77.9] | **0.001** | 189.1 [126.3 -216.4] | 37.2 [15.0 - 57.9] | **0.01** | |
| | 12 | 3.4 [3.4 - 3.4] [b] | 4.2 [3.4 - 6.8] | 1.2 [1.0 - 2.0] | 0.12 | 76.1 [68.6 - 142.8] | 38.8 [22.1-42.0] | **0.01** | 116.7 [57.3 - 205.0] | 20.3 [15.1 - 60.3] | **0.001** | 86.7 [55.8 - 173.2] | 25.5 [16.4 - 50.9] | **0.01** | |
| IL-10 | 2 | 13.2 [8.5 - 14.2] | N/A | N/A | N/A | 14.7 [8.1 - 38.0] | 1.0 [1.0 - 2.8] | 0.25 | 10.9 [9.0 - 32.2] | 1.3 [0.8 - 2.0] | 0.21 | N/A | N/A | N/A | 5.4 [3.8 - 30.6] |
| | 4 | 13.6 [8.9 - 18.3] | 14.0 [7.6 - 15.3] | 0.8 [0.7 - 2.0] | 0.67 | 20.5 [18.7 - 26.5] | 1.4 [0.9 - 2.0] | 0.06 | 16.3 [12.6 - 27.3] | 1.7 [1.0 - 1.9] | **0.01** | 14.2 [11.4 - 18.6] | 1.1 [0.7 - 1.6] | 0.56 | |
| | 12 | 9.7 [4.6 - 12.3] | 7.2 [5.4 - 13.7] | 1.3 [1.0 - 1.5] | 0.25 | 12.2 [10.3 - 16.1] | 1.4 [0.8 - 1.7] | 0.06 | 14.0 [9.0 - 19.0] | 1.8 [1.1 - 2.4] | **0.01** | 8.6 [7.7 - 11.1] | 1.4 [0.8 - 2.0] | 0.15 | |
