# Supplementary material for: Seminal plasma induces inflammation and enhances HIV-1 replication in human cervical tissue explants
Source: PLoS Pathog. 2017 May 19;13(5):e1006402. doi: 10.1371/journal.ppat.1006402 (PMC5453613; doi:10.1371/journal.ppat.1006402)
Supplement: S2 Table — N-fold change in gene expression measured in ectocervical explants incubated with seminal plasma (SP) 50% and/or indomethacin 10μM for 4 and 12 h, compared to donor-matched untreated explants (CM). Values are reported as median and IQR (n = 7). P<0.05 (in bold) indicates a statistically significant difference with CM (Wilcoxon signed rank test). (PDF) [file ppat.1006402.s011.pdf]

		CM + indomethacin		SP50%		SP50% + indomethacin	
Gene	Incubation time (h)	N-fold (CM)	p-value	N-fold (CM)	p-value	N-fold (CM)	p-value
IL1A	4	1.1 [0.3 - 1.2]	0.93	4.2 [1.9 - 5.8]	**0.03**	2.6 [1.4 - 5.4]	**0.03**
	12	0.8 [0.5 - 1.1]	0.56	7.0 [2.7 - 16.9]	**0.01**	11.1 [2.4 - 16.1]	**0.01**
IL6	4	0.5 [0.3 -1.1]	0.29	2.6 [1.8 - 5.5]	**0.03**	2.4 [2.0 - 4.5]	**0.01**
	12	1.3 [0.4 - 1.9]	0.68	4.2 [3.5 - 6.4]	**0.01**	1.8 [1.5 - 5.0]	**0.03**
TNF	4	0.7 [0.4 - 1.1]	0.20	2.9 [1.0 - 5.7]	0.06	1.4 [0.9 - 2.8]	0.12
	12	1.2 [0.7 - 1.5]	0.55	6.1 [3.4 - 17.1]	**0.03**	10.2 [3.1 - 19.8]	**0.01**
CCL5	4	0.7 [0.6 - 0.9]	**0.03**	0.5 [0.3 - 0.6]	0.27	0.6 [0.3 - 0.8]	0.15
	12	0.9 [0.6 - 1.7]	1.00	1.0 [0.6 - 1.7]	0.84	0.9 [0.7 - 1.6]	0.80
CCL20	4	0.5 [0.3 - 0.9]	**0.04**	4.5 [1.7 - 19.2]	**0.01**	5.6 [2.2 - 10.6]	**0.01**
	12	0.6 [0.3 - 2.6]	0.93	4.2 [7.2 - 18.9]	**0.01**	10.3 [1.9 -14.9]	**0.01**
CXCL1	4	0.5 [0.3 - 1.0]	0.10	1.3 [0.6 - 2.2]	0.35	1.4 [1.1 - 3.0]	0.07
	12	2.0 [0.7 - 3.6]	0.11	4.6 [2.3 - 7.6]	**0.01**	4.7 [1.8 - 8.0]	**0.03**
CXCL8	4	0.7 [0.4 - 1.1]	0.20	1.7 [1.1 - 4.4]	**0.01**	1.8 [1.4 - 3.7]	**0.01**
	12	1.4 [0.6 - 2.2]	0.23	5.3 [3.1 - 9.6]	**0.01**	4.6 [2.5 - 17.3]	**0.01**
TGFB1	4	1.3 [0.9 - 1.9]	0.17	0.5 [0.4 - 1.1]	0.14	0.7 [0.2 - 0.8]	**0.03**
	12	1.0 [0.6 - 1.3]	0.83	0.4 [0.3 - 1.0]	0.40	0.6 [0.4 - 0.9]	0.12
IL10	4	0.7 [0.6 - 0.8]	**0.02**	0.6 [0.4 - 0.8]	0.05	0.6 [0.4 - 0.8]	**0.02**
	12	0.8 [0.6 - 1.3]	0.55	1.1 [0.5 - 1.5]	0.67	0.8 [0.6 - 1.2]	0.46
CSF2	4	0.9 [0.3 - 1.3]	0.55	1.5 [1.0 - 5.0]	0.18	1.4 [0.9 - 1.6]	0.23
	12	0.9 [0.3 - 1.8]	0.86	2.7 [1.5 - 7.3]	**0.03**	3.3 [1.9 - 9.8]	**0.03**
IL7	4	0.7 [0.6 - 0.9]	0.05	2.4 [1.0 - 3.7]	0.06	1.6 [0.8 - 3.8]	0.15
	12	0.9 [0.7 - 1.4]	0.80	8.5 [4.0 - 36.7]	**0.01**	15.2 [2.6 - 35.1]	**0.01**
PTGS2	4	0.6 [0.4 - 0.8]	0.08	1.7 [1.1 - 1.9]	0.06	1.3 [1.1 - 1.7]	**0.03**
	12	1.5 [0.4 - 1.6]	0.66	2.7 [1.6 - 4.1]	**0.02**	2.3 [2.2 - 4.0]	**0.03**
