# Supplementary material for: Seminal plasma induces inflammation and enhances HIV-1 replication in human cervical tissue explants
Source: PLoS Pathog. 2017 May 19;13(5):e1006402. doi: 10.1371/journal.ppat.1006402 (PMC5453613; doi:10.1371/journal.ppat.1006402)
Supplement: S3 Table — N/A not applicable. (PDF) [file ppat.1006402.s012.pdf]

Gene	Sequence (5ˡ – 3ˡ)	Amplicon size (bp)	GeneBank reference	Publication reference
ACTB	CGTGATGGTGGGCATGGGTC<br>ACACGCAGCTCATTGTA	162	NM_001101.3	[20]
GAPDH	CGGATTTGGTCGTATTGGG<br>GCTTCCCGTTCTCAGCCTTG	173	NM_002046.5	N/A
UBC	GAGCGGAACAGGCGAGGAAA<br>GAACTGCGACCCAAATCCCG	145	NM_021009.6	N/A
IL1A	CCAACGGGAAGGTTCTGAAG<br>GGCGTCATTCAGGATGAATTC	191	NM_000575.3	[20]
IL6	GGTGTTGCCTGCTGCCTTC<br>GCCAGTGCCTCTTTGCTGCT	197	NM_000600.3	N/A
TNF	AGGCGGTGCTTGTTCCTCAG<br>AGGCTTGTCACTCGGGGTTC	183	NM_000594.3	N/A
CCL5	CTCGCTGTCATCCTCATTGCT<br>TGTGGTGTCCGAGGAATATGG	62	AF043341	[20]
CCL20	TTGCTCCTGGCTGCTTTG<br>ACCCTCCATGATGTGCAAG	364	BC020698.1	[54]
CXCL1	CTGAGGAGCCTGCAACATGC<br>TGCACATACATTCCCCTGCCT	98	NM_001511.3	N/A
CXCL8	GCCAGGAAGAAACCACCGGAA<br>AAACTGCACCTTCACACAGAGC	118	NM_000584.3	N/A
TGFB1	GTTGTGCGGCAGTGGTTGAG<br>GCCGGTAGTGAACCCGTTGAT	120	NM_000660.5	N/A
IL10	TCCCAGGCAACCTGCCTAAC<br>AGGCTTGGCAACCCAGGTAA	150	NM_000572.2	N/A
CSF2	AGCCCTGGGAGCATGTGAAT<br>CGGCTCCTGGAGGTCAAACA	125	NM_000758.3	N/A
IL7	GGCGTGGGTAAGAGGAACCA<br>TGATGACCGCAACTGGAGCA	131	J04156.1	N/A
PTGS2	TGCCTGATGATTGCCCGACT<br>TGAAAGCTGGCCCTCGCTTA	198	NM_000963.1	N/A
HIV-1 *pol*	TACAGGAGCAGATGATACAG<br>CCTGGCTTTAATTTTACTGG	267	NC_001802.1	[76]
HBB	GCTTCTGACACAACTGTGTTCACTAGC<br>CACCAACTTCATCCACGTTCACC	120	NG_000007.3	[77]
